# Small-molecule metabolome identifies potential therapeutic targets against COVID-19

**DOI:** 10.1038/s41598-022-14050-y

**Published:** 2022-06-15

**Authors:** Sean Bennet, Martin Kaufmann, Kaede Takami, Calvin Sjaarda, Katya Douchant, Emily Moslinger, Henry Wong, David E. Reed, Anne K. Ellis, Stephen Vanner, Robert I. Colautti, Prameet M. Sheth

**Affiliations:** 1 Gastrointestinal Diseases Research Unit (GIDRU), Kingston Health Sciences Centre, 76 Stuart St., Kingston, ON K7L 2V7 Canada; 2 Department of Psychiatry, Queen’s University, Kingston, ON Canada; 3 Division of Microbiology, Kingston Health Sciences Centre, Kingston, ON Canada; 4 Division of Allergy and Immunology, Department of Medicine, Queen’s University, Kingston, ON Canada; 5 Department of Biology, Queen’s University, Kingston, ON Canada; 6 Department of Pathology and Molecular Medicine, Queen’s University, Kingston, ON Canada

**Keywords:** Scientific data, Viral infection, Biomarkers, Mass spectrometry

## Abstract

Respiratory viruses are transmitted and acquired via the nasal mucosa, and thereby may influence the nasal metabolome composed of biochemical products produced by both host cells and microbes. Studies of the nasal metabolome demonstrate virus-specific changes that sometimes correlate with viral load and disease severity. Here, we evaluate the nasopharyngeal metabolome of COVID-19 infected individuals and report several small molecules that may be used as potential therapeutic targets. Specimens were tested by qRT-PCR with target primers for three viruses: Influenza A (INFA), respiratory syncytial virus (RSV), and SARS-CoV-2, along with unaffected controls. The nasopharyngeal metabolome was characterized using an LC–MS/MS-based screening kit capable of quantifying 141 analytes. A machine learning model identified 28 discriminating analytes and correctly categorized patients with a viral infection with an accuracy of 96% (R^2^ = 0.771, Q^2^ = 0.72). A second model identified 5 analytes to differentiate COVID19-infected patients from those with INFA or RSV with an accuracy of 85% (R^2^ = 0.442, Q^2^ = 0.301). Specifically, Lysophosphatidylcholines-a-C18:2 (LysoPCaC18:2) concentration was significantly increased in COVID19 patients (*P* < 0.0001), whereas beta-hydroxybutyric acid, Methionine sulfoxide, succinic acid, and carnosine concentrations were significantly decreased (*P* < 0.0001). This study demonstrates that COVID19 infection results in a unique nasopharyngeal metabolomic signature with carnosine and LysoPCaC18:2 as potential therapeutic targets.

## Introduction

COVID-19 represents one of the greatest public health challenges of the twenty-first century. Unlike most respiratory viruses, SARS-CoV-2 has a longer incubation period and infected individuals present with a spectrum of symptoms ranging from asymptomatic to severe clinical disease requiring hospitalization. The majority of SARS-CoV-2 infections occur via the nasal mucosa^1^. Understanding the host–pathogen interactions in the nasal mucosa may provide valuable insight into the identification of novel therapeutic targets. These targets may be used to interrupt the acquisition and limit disease progression of SARS-CoV-2. We thus examined if the nasal metabolomic profile for COVID-19 was distinct from those of other respiratory viruses, and whether examining the metabolome of the nasopharynx (NP) would provide insight into host–pathogen interactions in the nasal mucosa.

Previous studies involving the nasal metabolome in individuals infected with respiratory viruses, including rhinovirus (RV) and respiratory syncytial virus (RSV), reported that the nasal metabolome was virus-specific, despite indistinguishable clinical presentations in infected individuals^2^. Furthermore, the concentrations of specific nasal metabolites positively correlated with viral load and disease severity and predicted the need for positive pressure ventilation in patients with a high degree of sensitivity and specificity (84% and 86%, respectively)^3^. The predominant changes in the nasal metabolome observed in response to respiratory viruses were identified to be host-derived, although some metabolite concentrations correlated with colonization with *Haemophilus influenzae*, *Streptococcus pneumoniae* and *Moraxella catarrhalis*^2^. These studies suggest that evaluating metabolic signatures in the nasopharynx of COVID-19 patients compared to other respiratory viruses may provide insight into important host-mediated antiviral responses, further elucidate changes that may be occurring in the nasal microbial environment and potentially identify new therapeutic targets against COVID-19.

**Figure 1:**
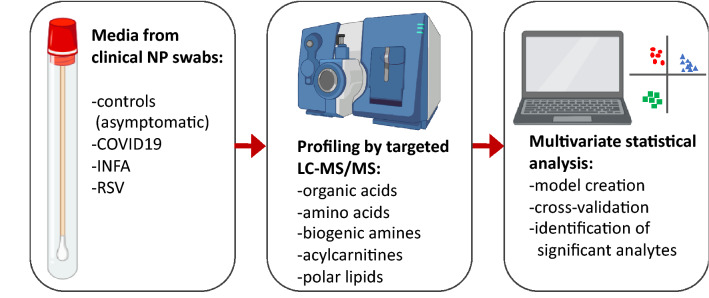
Experimental workflow. Viral transport medium from clinical nasopharyngeal swabs was analyzed using a TMIC Prime kit involving chemical derivatization, and liquid chromatography-tandem mass spectrometry. Multivariate and univariate statistical analyses were conducted to identify significant analytes, which we attempt to rationalize in the context of the pathogenesis of viral infection.

In this study we hypothesized that SARS-CoV-2 induces a characteristic change to the nasal metabolome of patients and that could be used to both identify metabolites important in pathogenicity, as well as potential therapeutic targets. We thus implemented a targeted metabolomics approach based on liquid chromatography tandem mass spectrometry (LC–MS/MS) to (1) characterize small-molecule profiles in viral transport media (VTM) from NP swabs of patients infected with INFA, RSV or COVID-19 and unaffected controls; (2) identify COVID-19 specific metabolite patterns; and (3) explore potential therapeutic pathways based on significant metabolites identified by a supervised machine learning model (Fig. 1).

## Results

### Small molecule profiling of NP swabs from patients with respiratory infection

We studied the nasopharyngeal metabolome in patients who underwent standard-of-care or screen testing for respiratory infection. VTM from clinical samples were analyzed using a targeted, small-molecule screening kit (TMIC Prime) capable of quantifying 141-analytes over six chemical classes using a combination of LC–MS/MS and flow-injection analysis-MS/MS (Fig. 1). A total of 210 individuals were included in this study, comprising 44 unaffected controls (AC) and three patient groups: 55 patients positive for SARS-CoV-2 (COV), 55 patients positive for INFA and 56 positive for RSV (Table 1). PCR Cycle Threshold (CT) values were used as a surrogate for viral load (VL) and samples were stratified according to CT’s of 30–35 (low VL), 25–30 (intermediate VL) and < 24.9 (high VL).

**Table 1:** Patient demographics.

	Patient group			
	SARS-COV-2 (COV)	Influenza A (INFA)	Respiratory Syncytial Virus (RSV)	Unaffected Controls
N	55	55	56	44
Year of collection (range in months)	2020 (Jan–Apr)	2019–2020 (Dec–Mar)	2019–2020 (Dec–Mar)	2020 (Sept)
Median age (range)	55 years (20–85 years)	62 years (27 days–94 years)	16 months (21 days–91 years)	24 years (19–43 years)
Sex (%)	M 27% F 42% n/a 31%	M 54% F 44% n/a 2%	M 40% F 46% n/a 14%	M 25% F 75%
Median CT^a^ (range)	26.2 (17.76–37.24)	28.6 (21.43–38.6)	26.3 (18.54–36.87)	N/A

^a^PCR cycle threshold values (CT).

As the TMIC Prime kit was developed for profiling biological matrices such as serum, urine and stool, we first surveyed which analytes could be detected in VTM from clinical NP swabs. All patients and subjects were sampled using nasopharyngeal swab kits from the same manufacturer (Copan Diagnostics, USA). The mean concentration of 46 of the 141 analytes measured was found to be at least 2X greater in all clinical samples, as compared with blank VTM obtained from unused swab kits. Analytes comprised amino acids (N = 18), organic acids/derivatives (N = 5), biogenic amines (N = 11) acylcarnitines (N = 2), polar lipids (N = 8), sphingomyelins (N = 1), and organic nitrogen-containing compounds (N = 1); suggesting that a range of analytes can be sampled from the nasopharynx and then recovered from VTM (Supplementary Table S1).

When comparing all patient samples (SARS-CoV-2, INFA and RSV) with unaffected controls, the feature selection step of our machine learning pipeline identified a subset of 28 significant metabolites which differentiated patients and controls. This method was also used to identify 5 metabolites that distinguished SARS-CoV-2 from Influenza and RSV, resulting in 30 unique metabolites that we prioritized for multivariate analysis, including amino acids (N = 15), organic acids (N = 4), acylcarnitines (N = 1), polar lipids (N = 4), biogenic amines (N = 5) and total hexoses (N = 1) (Supplementary Tables S1 and S2).

### Multivariate model creation and testing

**Figure 2:**
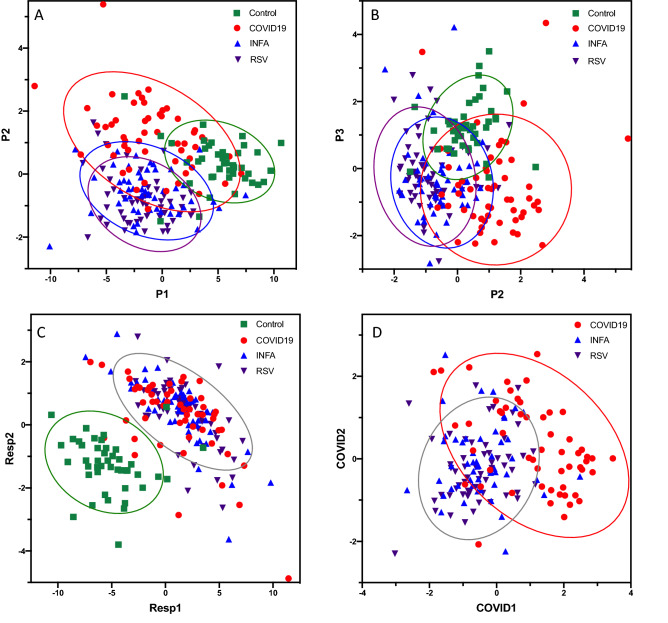
Multivariate analysis and classification of patients with respiratory illness based on metabolite profiles. Supervised partial least squares discriminant analysis was used to plot analyte profiles in VTM from clinical nasopharyngeal swabs scaled to control VTM. Plots of components 1 and 2 (**A**) and 1 and 3 (**B**) are shown where optimal separation of patient groups was observed. Orthogonal partial least squares discriminant analysis was used to plot analyte profiles among patient groups. In (**C**), all patients with a respiratory illness were grouped into a single category and compared to unaffected controls. In (**D**), COVID19 patients were compared to all other patients with influenza A and RSV were tr into a single category. The 95% confidence region is circled for each category.

**Figure 3:**
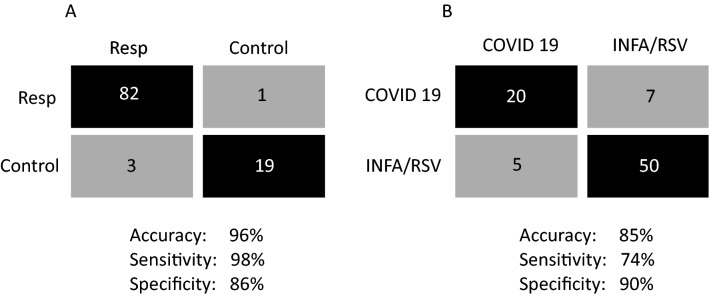
Confusion matrices based on test/train cohorts using 50% of the data. Shown in (**A**) and (**B**) from which the accuracy, sensitivity and specificity of identifying patients with a respiratory infection in general (**A**) or patients with COVID19 among patients with respiratory illness (**B**) was determined.

Exploratory modelling of metabolite profiles scaled to blank VTM using partial-least discriminant analysis (PLS-DA) revealed some separation of the four patient groups (Fig. 2A, B). A second model using orthogonal partial least squares discriminant analysis (OPLS-DA) focused on differences between all patients with respiratory illness (SARS-CoV-2, INFA and RSV) and unaffected controls, where the two groups appeared well-separated (Fig. 2C). Using half of the data for training and the other half for testing, this model had an accuracy of 96%, a sensitivity of 98% and specificity of 86% (R^2^ = 0.771, Q^2^ = 0.72) in differentiating between groups (Fig. 3A). Both training and testing sets exhibited area-under the curve measurements based on receiver operating characteristic curves of > 0.90, shown in the Supplementary Methods (AUROC; training = 0.98, testing = 0.91). A third model compared COVID19 patients and those with other respiratory illnesses (INFA or RSV) (Fig. 2D) Using the same cross-validation analysis as above, this model distinguished COVID19 from other patients with an accuracy of 85%, sensitivity of 74% and specificity of 90% (R^2^ = 0.442, Q^2^ = 0.301) (Fig. 3B). AUROC measurements for both training and testing sets were similar (AUROC training = 0.85, testing = 0.84, Supplementary Methods).

### Five analytes are specifically altered in patients with SARS-CoV-2 infection

**Figure 4:**
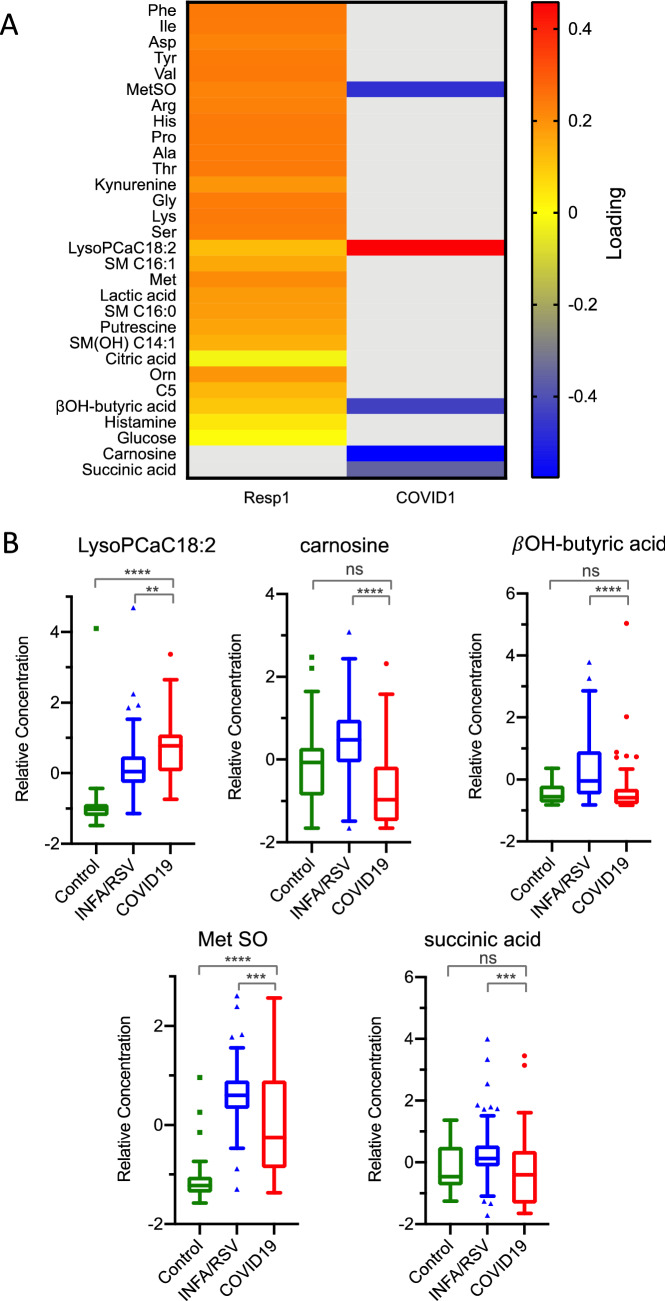
Feature selection from OPLS-DA models. (**A**) Metabolite loadings for respiratory infection and COVID19 models highlighting the most significant features. For the respiratory infection model, the heatmap denotes the relative association of each metabolite with respect to unaffected controls. For the COVID19 model, the heatmap denotes the relative association of each metabolite with respect to influenza A/RSV patient group. (**B**) Boxplots showing relative concentrations of significant metabolites from the COVID19 model are mean-centered at zero. For metabolites presented in (**B**), *P* < 0.0001 by Kruskal–Wallis test. Significance of between-group means by post hoc Dunn’s test is given in each plot.

OPLS-DA loadings for the 30 unique metabolites that differed significantly among patient groups and controls are shown in Fig. 4A. Twenty-eight analytes exhibited increased concentrations in patients with respiratory infections as compared with unaffected controls, including amino acids, polar lipids, organic acids, and biogenic amines. Most importantly, a smaller subset of analytes was observed to be specifically increased (LysoPCaC18:2) or decreased (MetSO, beta hydroxy-butyric acid, carnosine, and succinic acid) in COVID19 patients as compared with INFA or RSV (Fig. 4B). Interestingly, carnosine and succinic acid were not found to be important factors in differentiating all respiratory patients from controls (Fig. 4A, B).

Despite their ability to distinguish among patient groups, none of these five metabolites correlated significantly with VL (qRT-PCR CT) for any of the three respiratory viruses (Supplementary Fig. S1). There is a lack of consensus regarding the correlation between CT and COVID19 disease severity, particularly in non-hospitalized patients^4^. Our COVID19 patients were all symptomatic, and sampled during acute phase of infection, but were not considered severe and did not require hospitalization. Our INFA and RSV cohorts likely exhibited a range of severities as they were sampled in a variety of clinical settings such as outpatient clinics, emergency departments and upon hospital admission. We were unable to assess the relative severity of symptoms in our INFA and RSV cohorts, as there was no quantifiable severity scale for these infections. While biological sex did not appear to have an effect on metabolite profiles, we must acknowledge the possibility that age had an effect, as median age in the control group (24 years) was lower than in COVID19 (55 years) or INFA (62 years) and the RSV group had the lowest median age of 16 months (Table 1, and Supplementary Figure S2 and Supplementary Methods). We conclude that age likely had minimal impact on metabolite profiles in the Resp and COVID models, as RSV patients were grouped with either INFA and/or COVID19. Furthermore, when treated as individual classes, INFA and RSV metabolite profiles overlapped despite having different age profiles, and minimal overlap occurred between the younger control and RSV cohorts (Fig. 2A, B).

## Discussion

Using targeted LC–MS/MS-based metabolomics, we identified unique metabolite profiles associated with the nasopharynx of patients with common respiratory infections. We observed striking differences in signatures that could be used to differentiate unaffected controls from patients with COVID19, INFA or RSV. Furthermore, we identified a COVID-19-specific signature, characterized by altered concentrations of LysoPCaC18:2, beta-hydroxybutyric acid, Met SO, succinic acid, and carnosine, relative to INFA and RSV.

While several metabolomics studies related to COVID-19 have emerged, the use of both targeted and untargeted approaches applied to a range of biosamples makes comparing results among studies challenging^5^. The current study is unique as it employed a targeted approach to profile VTM acquired from standard-of-care swab kits from a diverse cohort of patients with qRT-PCR-confirmed COVID-19, IFNA or RSV, as well as unaffected controls. Although two previous studies have analyzed NP swabs, one study assessed VTM using matrix-assisted laser desorption/ionization mass spectrometry (MALDI-TOF MS)^6^. The other study analyzed fresh swabs directly by ambient ionization methods including DESI and LD-REIMS and focused on lipid profiling^7^. Both studies revealed diagnostic accuracies of > 80%. At least three studies investigating the serum metabolome of COVID-19 patients identified changes in the tryptophan-kynurenine pathway associated with regulation of inflammation^8^. We also observed an increase in kynurenine concentration in our model comparing patients with all respiratory diseases with controls, but this metabolite was not COVID-19 specific. Our results are consistent with Shen et al*.*^9^ who also observed increased kynurenine concentration in the serum of non-COVID-19 patients presenting with symptoms of respiratory infection. Amino acids are decreased in interleukin-6 stratified COVID-19 patients compared to controls purportedly due to renal dysfunctional and marked alterations in nitrogen metabolism^8^. In contrast, we saw an increase in amino acids in respiratory virus infection compared to control, yet with the above studies including Shen et al*.*, this may reflect a difference in systemic (serum) vs local (nasopharynx) compartments sampled. Of the three studies involving serum metabolome analysis, Blasco et al*.*^10^ calculated a diagnostic accuracy for COVID-19 as 74%.

**Figure 5:**
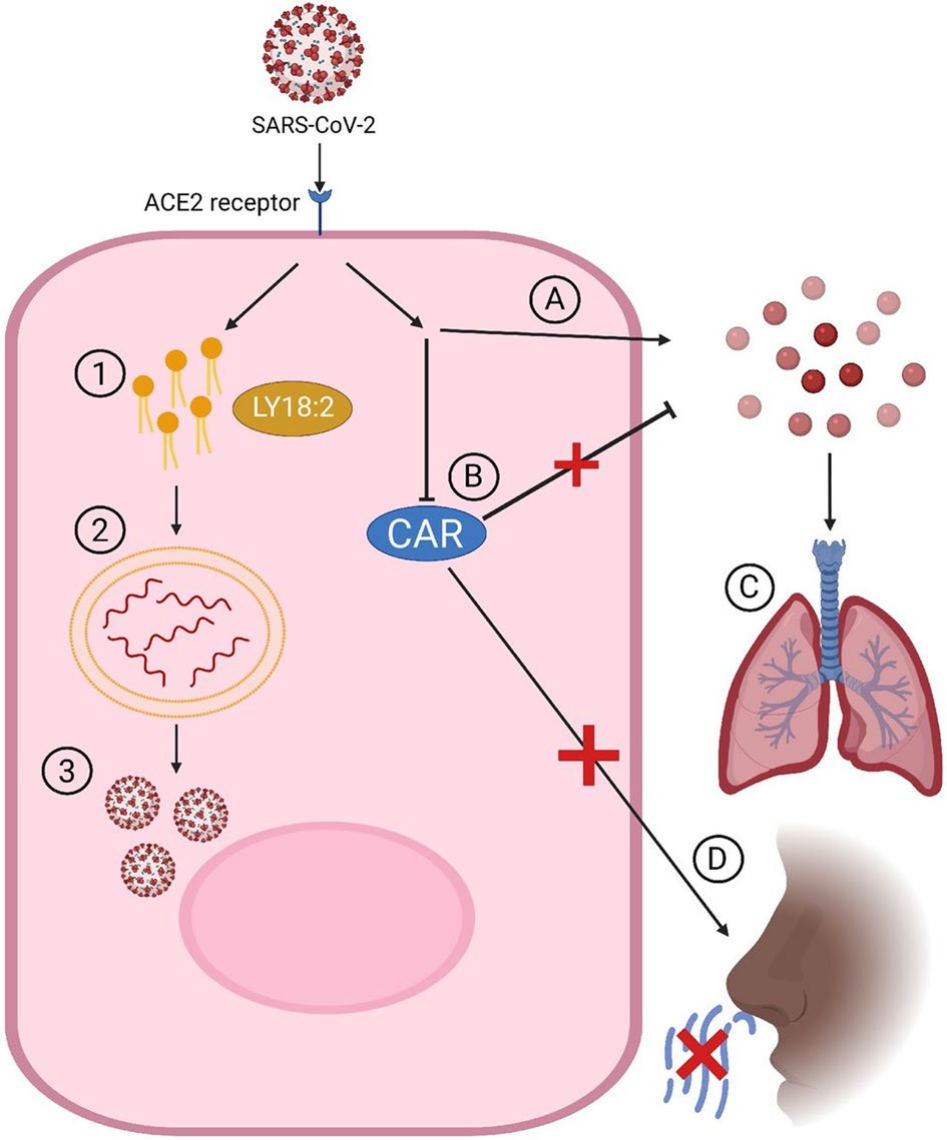
Schematic view of SARS-CoV-2 infection and potential mechanisms of symptom generation involving significantly altered metabolites. (**1**) After viral entry into the cell an increase in lipid generation occurs through viral hijacking of cell machinery. (**2**) Lipids are used to generate double membrane vesicles for replication. (**3**) Release of new Coronavirus. (**A**) SARS-CoV-2 leads to an elevation in oxidative stress by generation of reactive oxygen species (ROS). (**B**) Decreased levels of Carnosine results in a reduced ability for antioxidant clearance of ROS. (**C**) Oxidative stress and inflammation can damage the lungs and lead to further symptoms of COVID-19. (**D**) Reduction of Carnosine within the cells of the olfactory may be implicated in anosmia, a common symptom of COVID-19.

The COVID-19-specific metabolomic profile yields potential insights into the mechanism of infection of SARS-CoV-2 (Fig. 5). Carnosine and LysoPCaC18:2 had strong loadings in the OPLS-DA model, with the former decreasing, and the latter increasing, in COVID-19 patients relative to patients with INFA or RSV. Carnosine, a naturally occurring dipeptide, has a wide range of protective effects in humans, which are largely attributed to its powerful antioxidant actions^11^. Several mechanisms could explain the depleted levels of carnosine in COVID-19 patients. First, decreased carnosine levels may signify decreased production of the dipeptide by the host. The olfactory system is among the richest sources of carnosine in humans^12,13^, and carnosine present in the nasal swabs likely originated from the olfactory epithelium at the roof of the nasal cavity. The downregulation of carnosine could reflect decreased biosynthesis/secretion by olfactory sensory nerves or progressive loss of these neurons. Second, reduced carnosine levels may be the result of increased dipeptide degradation. Carnosine is largely metabolized by carnosinase-1 (CN1)^11^. Although CN1 is expressed by the human olfactory epithelium^14^, the nasal cavity is not typically considered a site of high carnosinase activity, such that intranasal administration of carnosine has been employed in a preclinical model of Parkinson disease as a means to avoid degradation by carnosinase^15^. A third, and more probable explanation, is that the diminished carnosine levels indicate depleted carnosine stores. Our data show that even basal levels of the dipeptide are completely exhausted in COVID-19 patients. Saadah et al*.*^16^ predicted that COVID-19-induced oxidative stress would result in carnosine depletion. The protective effects of carnosine are widely attributed to its antioxidant, anti-glycation, and anti-inflammatory properties^11^. For instance, reduced circulating levels of low-density lipoprotein (LDL) has been associated with increased risk of acute kidney injury in COVID-19 patients^17^, and carnosine, known to block lipid peroxynitrite-mediated modification of human LDL at physiological levels^18^, may protect against LDL degradation. Intriguingly, recent studies suggest that carnosine may also protect against SARS-CoV-2 infection through more specific mechanisms. Molecular docking and modelling studies identified carnosine as the most promising drug candidate to prevent the binding of SARS-CoV-2 to the ACE2 receptor^16^. Sustentacular cells of the olfactory system co-express the ACE2 receptor as well as TMPRSS2, a protease that facilitates viral entry, making these cells highly susceptible to SARS-CoV-2^19^ . Infection of these cells has been implicated in anosmia^9^, a recognized symptom of COVID-19^20^. Given that the olfactory epithelium is a major producer of carnosine and this dipeptide’s vital neuroprotective role in this system^21^, loss of carnosine may lead to olfactory nerve damage resulting in anosmia in COVID-19.

Severe cases of COVID-19 are associated with multi-organ damage arising from oxidative stress^22^, raising the possibility of global depletion of carnosine in these patients and underscoring its therapeutic potential^23^. Recently, Kulikova et al.^24^ developed an analogue of carnosine, salicyl-carnosine, designed to circumvent the rapid degradation by serum carosinase. Salicyl-carnosine effectively mitigates the three prominent pathogenic hallmarks of COVID-19—oxidative stress, thrombosis, and inflammation—and has been proposed as a promising means to treat severe cases of COVID-19^25^.

Lysophosphatidylcholine (LysoPCaC18:2) is an endogenous bioactive phospholipid suggested to be important in coronavirus infection. The hijacking of host cells by the virus to create a proper environment for replication involves the generation of specialized vesicles of which LysoPCaC18:2 is required^26^. Our study identified significantly elevated levels of LysoPCaC18:2 in COVID19 positive samples compared to INFA and RSV. In COVID19 viral infection, a change towards an increase in lipid generation has been demonstrated and further supports our finding^27,28^ especially whereby LysoPCaC18:2 was higher in COVID19 samples compared to healthy controls^29^. Additionally, a study inhibiting Cytosolic Phospholipase A2α (cPLA2α) which produces lysophospholipids, had significant effects on lowering coronavirus RNA and protein accumulation due to the importance of phospholipids in the creation of replicative organelles, emphasizing the therapeutic potential of lipid metabolism pathways. Moreover, the same study found that inhibition of cPLA2α had no impact on the replication of influenza A thus, consistent with the COVID19-specific pattern of LysoPCaC18:2 concentration observed in our study^30^.

During fasting or glucose depletion, beta-hydroxybutyric acid serves as the primary energy source to peripheral tissues, including the heart, brain, and muscles^31^. It also acts as a signaling molecule, participating in neural protection, lipid metabolism and gene expression^31–33^. Contrary to our results based on NP fluid, others studies have observed increased beta-hydroxybutyric acid in the serum of COVID-19 patients^34–36^. In each of those studies, beta-hydroxybutyric acid was elevated in patients with severe COVID-19, such that Shi et al. concluded that increased serum beta-hydroxybutyric acid predicted progression from mild to more severe disease^34^. In contrast, the COVID-19 cases in our study were not considered to be severe. The health benefits of beta-hydroxybutyric acid have prompted researchers to advocate for metabolic therapies that raise beta-hydroxybutyric acid levels to treat severe COVID-19^37,38^; as beta-hydroxybutyric acid was found to reduce coronavirus-dependent inflammation in mice^39^ and, protect COVID-19 antibodies against degradation through a post-translational modification^40^. To address the discordance between the therapeutic potential of beta-hydroxybutyric acid and its association with worsening COVID-19 disease, we speculate that in the acute phase of COVID-19, the liver synthesizes beta-hydroxybutyric acid, in part, to replenish depleted energy stores; however, as the patient deteriorates, liver function becomes impaired^36^, resulting in dysregulated overproduction of beta-hydroxybutyric acid and other ketone bodies.

We observed a decrease in levels of MetSO, a product of oxidative stress^41^ in patients with COVID-19 compared to those with Influenza and RSV. While previous studies have documented an increase in MetSO in the serum and plasma of COVID-19 patients compared to healthy controls^8,42–44^ there have been no studies prior to ours comparing such levels between COVID-19, Influenza and RSV respectively in the respiratory tract. In influenza an increase in pro-oxidative markers such as NAPDH oxidase occurs in infection and can cause severe lung injury^45^ while in RSV expression of antioxidant markers including catalase is decreased^46^. In one study by Olagnier et al. levels of Nuclear factor-erythroid 2 related factor 2 (NRF2), a protective antioxidant signaling was suppressed in both lung autopsies from patients with severe COVID-19 infection and an in-vitro infection model of SARS-COV-2^47^. These studies demonstrate that the host reaction to respiratory infection within the context of oxidative stress is arbitrated by the infecting virus. However, a study by Sharif-Askari et al. did not find significant difference between the expression of methionine sulfoxide reductase A between severe COVID-19 infection, influenza and RSV^48^. Studies regarding succinic acid in the context of COVID-19 in respect to other respiratory infection are lacking. Moreover while compared to healthy controls succinic acid was enriched in the serum of patients with COVID-19^34^ subjects who had recovered from COVID-19 infection but had a moderate and severe or critical illness 3 months after discharge had decreased plasma levels of succinic acid compared to healthy controls^49^. Further investigation will be needed to identify the significance of lower MetSO and succinic acid in COVID-19 infection.

A limitation of our study was that extensive validation of the TMIC Prime kit for use with VTM was not conducted, including evaluation of matrix effects. While recovery of most synthetic metabolites was demonstrated at a single level in VTM, accuracy and precision may have been affected by matrix interferences (Supplementary Table S2). Other aspects that should be evaluated include recovery of analytes from the swab, and how recoveries and metabolite profiles might differ when using swab kits from other manufacturers. For the purposes of this pilot study, we used TMIC Prime as a rapid screening method to evaluate broad metabolite profiles in patients with respiratory diseases that will form the basis of more refined assays for individual metabolites in future studies. Although broad, 141 metabolites screened within the nasopharynx is comparatively limited compared to other untargeted techniques, TMIC Prime has the advantage of being quantitative for most analytes, and the number of analytes screened could be extended in future studies. Our observation that only 46 analytes were detectable in VTM (out of 141) implies a unique composition of the NP metabolome as compared with other biological matrices that can be surveyed with TMIC Prime, but does not exclude the possibility that more small molecules are detectable with broader targeted or untargeted methods. Isotopically-labeled internal standards were used to correct for recovery of most analytes from VTM, but we were unable to specifically account for differences in yield of NP fluid sampled by each swab. As such, sum-normalization of analyte concentrations was employed to minimize the effect of variability in NP volume sampled. Furthermore, assay performance metrics for certain polar lipids such as LysoPCaC18:2 were not determined as we lacked a synthetic standard, and the internal standard used for this lipid was non-specific. Finally, while this study did not include patients with allergic rhinitis, it can be anticipated that the NP metabolome would be different from actual viral infection as different mechanisms are at play during allergy and viral infection and replication. However there are currently no studies which have investigated this specifically.

In conclusion, we demonstrated that the metabolome of the nasopharynx can be measured from clinical nasal swabs, and that metabolite profiles identified using machine learning methods can differentiate patients with COVID-19 from other respiratory virus infections (e.g. INFA/RSV). Our study identified key metabolites specifically altered in COVID19 such as carnosine and LysoPCaC18:2 that have previously been implicated in viral replication and symptom generation. This enables us to propose mechanisms contributing to viral infection and propagation as well as potential targets for COVID-19 therapy.

## Materials and methods

### Ethics

All experimental protocols were approved by and conducted in accordance with the Queen’s University Health Sciences and Affiliated Teaching Hospitals Research Ethics Board (HSREB Files 6029794, 6029811).

### Sample collection and qRT-PCR analysis

Symptomatic patients undergoing testing for SARS-CoV-2, INFA, and RSV at Kingston Health Sciences Centre (KHSC) and surrounding hospitals, were sampled using nasopharyngeal (NP) swabs which were then stored in viral transport media (Copan Diagnostics, USA). Unaffected, asymptomatic participants were recruited as controls and tested for SARS-CoV-2 as part of a surveillance study testing medical and nursing students during the 2020 SARS-CoV-2 lockdown in Canada. All COVID19 patients in our study were symptomatic, and sampled during the acute phase of the infection. Our COVID19 cohort were not considered to have severe illness, and did not require hospitalization. Enrolled INFA and RSV patients were swabbed in a combination of clinical settings, including out-patient clinics, acute-care emergency departments or upon admission to hospital, and thus likely exhibited a range of symptom severities.

Total RNA was extracted from VTM on an automated nucleic acid extractor (Maxwell RSC 16) using a Maxwell RSC whole blood RNA/DNA kit (Promega, Madison, WI). Presence of SARS-CoV-2 was tested using a multiplex quantitative real-time PCR (qRT-PCR) assay, targeting the envelope (E) and RNA-dependent RNA polymerase (RdRp) genes as described previously^50^. Samples assayed for INFA and RSV were obtained in 2019–2020 prior detection of SARS-CoV-2 in Canada and stored at − 80 °C. Testing for INFA, and RSV was conducted using a clinically validated laboratory-developed multiplex qRT-PCR assay for INFA (matrix), and RSV (nucleoprotein).

Biological samples and demographic data for all patients were obtained within the circle-of-care. To ensure researchers were blinded to patient identity, samples were de-identified and anonymized, and only non-identifying data including age, biological sex and travel history were provided to researchers. The HSREB waived the requirement to obtain written informed consent, as the samples were acquired for purposes of clinical testing, and were de-identified and anonymized. In accordance with the Personal Health Information Protection Act of Ontario, all patients possess the right to withhold or withdraw their consent to access, utilize, or disclose their personal health information. Patients are not disadvantaged if they choose to decline participation.

### Metabolomic analysis

#### Sample preparation and LC–MS/MS analysis

Metabolite profiling kits (TMIC Prime) were acquired from The Metabolomics Innovation Centre (TMIC, Edmonton AB, Canada)^51,52^. The kit is capable of quantifying 141 analytes over six chemical classes using a combination of LC–MS/MS and flow-injection analysis (FIA)-MS/MS. Target analytes comprised organic acids, amino acids, biogenic amines, total hexoses, acylcarnitines and polar lipids (phosphatidylcholines (PC), lysophosphatidylcholines (LysoPC), sphingomyelins (SM), and hydroxy-sphingomyelins SM(OH)) (Supplementary Table 1). LC–MS/MS analysis was conducted using an ExionLC AC Series ultra-high-performance liquid chromatography system QTRAP 5500 mass spectrometer (Sciex Canada, Concord, ON, Canada) in electrospray ionization (ESI) mode using optimized settings and MRM transitions provided by the manufacturer of the assay kit. For water-soluble analytes, separations were conducted on an Eclipse XDB-C18 HPLC column (3.5um, 3.0X100mm; Agilent, CA, USA) protected by a standard guard cartridge system (SecurityGuard Phenomenex, CA, USA). For FIA of lipid-soluble analytes and glucose, samples were injected directly into the mass spectrometer via PEEK tubing.

50 µL of viral transport media (VTM) was supplemented with appropriate internal standards and treated with 150 µL of 50% ethanol, homogenized by vortex mixing and sonication, and pelleted by centrifugation. The supernatant was dried on an N_2_ evaporator and re-dissolved in 50% ethanol. To assay organic acids, samples were transferred to a deep-well 96-well plate. The following 3 solutions were added to each well for derivatization: (1) 25 µL of 250 mM 3-nitrophenylhydrazine prepared in 50% methanol (2) 25 µL of 150 mM 1-ethyl-3-(3-dimethylaminopropyl) carbolimide prepared in methanol (3) 25 µL of 7.5% pyridine prepared in 75% methanol. The plate was shaken at room temperature for 2 h. 375 µL of water was added, and the plate was shaken for 20 min at room temperature. 125 µL was transferred to a new plate and diluted with 375 µL of 50% methanol. For LC separation, 10 µL of the sample was injected into the LC–MS/MS system in negative ionization mode, using a flow rate of 300 µL/min where mobile phase A consisted of 0.01% formic acid (FA) in water, and mobile phase B consisted of 0.01% FA in methanol. The linear gradient elution profile for mobile phase B was: t = 0 min, 30%; t = 1.5 min, 30%; t = 12.5 min, 85%; t = 12.51 min, 100%.

For assay of amino acids, biogenic amines, and polar lipids, a second aliquot of VTM were prepared as described above. Samples were aliquoted onto a filter paper disc in each well of the 96-well filter plate. Samples were dried for 30 min on an N_2_ evaporator. For derivatization of amino acids and biogenic amines, a 5% solution of phenyl-isothiocyanate (PITC) was prepared in equal parts ethanol/pyridine/water. 50 µL of the 5% PITC solution was added to each filter paper. The plate was covered and incubated at room temperature for 20 min. The plate was dried for 90 min to remove excess liquid. 300 µL of 5 mM ammonium acetate in methanol was added to each well, and the plate was shaken at room temperature for 30 min to extract analytes from the filter paper. The extract was collected by centrifugation. For LC–MS/MS analysis of derivatized amino acids and biogenic amines, 62.5 µL of the extract was combined with 62.5 µL of water in a new 96-well plate and shaken. 10 µL of the sample was injected into the LC–MS/MS system in positive ionization mode using a flow rate of 500 µL/min, where mobile phase A consisted of 0.2% FA in water, and mobile phase B consisted of 0.2% FA in acetonitrile. The linear gradient elution profile for mobile phase B was: t = 0 min, 0%; t = 0.5 min, 0%; t = 5.6 min, 95%; t = 6.5 min, 95%. For FIA-MS/MS of underivatized glucose, acylcarnitines and polar lipids, 25 µL of the extract was combined with 125 µL of FIA buffer in a new plate. FIA buffer was prepared by adding 9 mL of 0.1% FA to 260 mL of methanol and was used as the sample diluent and mobile phase. 20 µL of the sample was injected for FIA analysis using the following flow rate profile: t = 0 min, 30 µL/min; t = 1.6 min, 30 µL/min; t = 2.4 min, 200 µL/min; t = 2.8, 200 µL/min. Two injections were conducted for FIA analysis: one in negative ionization mode for measurement of glucose, and a second injection in positive ionization mode for measurement of acylcarnitines and polar lipids.

Quantification of analytes measured by LC–MS/MS was based on isotope dilution and quadratic calibration lines for each analyte. Peak integration and analyte quantification were completed using Analyst 1.7 (Sciex). Analytes measured by FIA-MS/MS were quantified using a relative quantification approach using a single representative internal standard for each analyte class. Supplementary Table 2 presents assay performance parameters for prioritized metabolites measured by LC–MS/MS. Four quality control samples based on solution standards (QC 1–3) and a low-level spiked VTM sample were measured 3 times on each of 3 assay days. 89% of measurements in solution standards were within 20% of target values, and all but one analyte exhibited total Coefficient of variation (CVs) of < 20%. Total CVs for spiked VTM was more variable, with only 52% of analyte measurements exhibiting CVs of < 20%. Mean % differences from target concentration was + 28% (range − 14.3 to 112%). Poorer assay performance metrics in VTM are likely due to the presence of matrix interferences that we were unable to fully evaluate in the current study. Blank VTM from unused NP swab kits (4 samples on each of 3 assay days) was used as an additional control to assess apparent baseline concentrations of targeted analytes arising from either low levels of analytes present in the VTM, and/or matrix interferences (Supplementary Table S2). Baseline concentrations of most analytes in blank VTM were within the concentration range of the lowest two calibrators. Mean fold-change of analyte concentrations in clinical samples as compared with blank VTM are shown in Supplementary Table S2 for prioritized analytes.

### Statistical analysis

Our fully open and reproducible analysis pipeline, written in **R** (v 4.3), is available online (https://github.com/ColauttiLab/COVID-Metabolomics) and detailed in the Supplementary Methods. Briefly, we first scaled samples to baseline levels observed in VTM and then autoscaled each metabolite to a mean of zero and unit standard deviation. We used individual univariate models to test whether a metabolite differed among patients from the four different categories. We then imputed missing values (N = 87 of 7735) and randomly divided our data into a model-building test dataset (50% of data) and a model-testing validation dataset (50% of data) that was excluded from the model-building pipeline. Metabolites with significant differences among groups in the test dataset, after false-discovery rate adjustment, were included in multivariate partial least-squares discriminant analysis (PLS-DA) models as implemented by the *opls* function from the **ropls** package^53^. The accuracy, sensitivity, and specificity of the multivariate models were tested on the validation dataset. Statistical analysis of individual metabolites among patient groups (i.e. univariate models) were performed using a non-parametric ANOVA (Kruskal–Wallis) with a post hoc Dunn’s test. Differences in metabolite concentrations were determined to be significant if *P* < 0.05.

## Supplementary Information

Supplementary Information.

## Data Availability

Raw data, and data analysis pipeline written in R (v. 4.3) generated during the current study are available on the DRYAD database, (DOI: TBD) and Supplementary Methods. (Reviewer note: data and code/analysis to be archived are available at https://github.com/ColauttiLab/COVID-Metabolomics for reviewing purposes).
