# Supplementary material for: Small-molecule metabolome identifies potential therapeutic targets against COVID-19
Source: Sci Rep. 2022 Jun 15;12:10029. doi: 10.1038/s41598-022-14050-y (PMC9200216; doi:10.1038/s41598-022-14050-y)
Supplement: Supplementary file 1 — Supplementary Information. [file 41598_2022_14050_MOESM1_ESM.pdf]

Supplementary Information

Small molecule metabolome identifies potential therapeutic targets against COVID-19

Sean Bennet, Martin Kaufmann, Kaede Takami, Calvin Sjaarda, Katya Douchant, Emily Moslinger, Henry Wong, David E Reed, Anne K. Ellis, Stephen Vanner, Robert I. Colautti, Prameet M. Sheth

NOTE: Raw data and fully reproducible code for this project are available on GitHub: http://bit.ly/COVID-Metabolomics

Contents

Table S1: List of metabolites measured using the TMIC Prime kit 2

Figure S1: Correlation of CTs with selected metabolites from the COVID model 5

Figure S2: COVID19 model stratified by age and biological sex 6

Table S2: Assay performance parameters for prioritized analytes measured by LC-MS/MS 7

Supplementary Methods 8

Table S1: List of metabolites measured using the TMIC Prime kit

**Acylcarnitines**			
C0	Carnitine	C10:1	Decenoylcarnitine
C2	Acetylcarnitine	C10	Decanoylcarnitine
C3:1	Propenoylcarnitine	C12:1	Dodecenoylcarnitine
C3	Propionylcarnitine	C12	Dodecanoylcarnitine
C4:1	Butenylcarnitine	C14:2	Tetradecadienylcarnitine
C4	Butyrylcarnitine	**C14:1[1,2]**	**Tetradecenoylcarnitine**
C3OH	Hydroxypropionylcarnitine	C14	Tetradecanoylcarnitine
C5:1[3]	Tiglylcarnitine	C12DC	Dodecanedioylcarnitine
C5	Valerylcarnitine	C14:2OH	Hydroxytetradecadienylcarnitine
C4OH	Hydroxybutyrylcarnitine	C14:1OH	Hydroxytetradecenoylcarnitine
C6:1	Hexenoylcarnitine	C16:2	Hexadecadienylcarnitine
C6	Hexanoylcarnitine	C16:1	Hexadecenoylcarnitine
C5OH	Hydroxyvalerylcarnitine	C16	Hexadecanoylcarnitine
C5:1DC	Glutaconylcarnitin	C16:2OH	Hydroxyhexadecadienylcarnitine
C5DC	Glutarylcarnitine	**C16:1OH[2]**	**Hydroxyhexadecenoylcarnitine**
C8	Octanoylcarnitine	C16OH	Hydroxyhexadecanoylcarnitine
C5MDC	Methylglutarylcarnitine	C18:2	Octadecadienylcarnitine
C9	Nonaylcarnitine	C18:1	Octadecenoylcarnitine
C7DC	Pimelylcarnitine	C18	Octadecanoylcarnitine
C10:2	Decadienylcarnitine	C18:1OH	Hydroxyoctadecenoylcarnitine
**Amino Acids**			
**Ala[1,2,3]**	**Alanine**	**Lys[1,2,3]**	**Lysine**
Arg[1,3]	Arginine	**Met[1,2,3]**	**Methionine**
**Asn[1,2]**	**Asparagine**	**Orn[1,2,3]**	**Ornithine**
**Asp[1,2,3]**	**Aspartate**	**Phe[1,2,3]**	**Phenylalanine**
**Cit[1,2]**	**Citrulline**	**Pro[1,2,3]**	**Proline**
**Gln[1,2]**	**Glutamine**	**Ser[1,2,3]**	**Serine**
Glu	Glutamate	**Thr[1,2,3]**	**Threonine**
**Gly[1,2,3]**	**Glycine**	Trp	Tryptophan
**His[1,2,3]**	**Histidine**	**Tyr[1,2,3]**	**Tyrosine**
**Ile[1,2,3]**	**Isoleucine**	**Val[1,2,3]**	**Valine**
**Leu[1,2]**	**Leucine**		
**Benzenoids**			
Tyramine		Homovanillic acid	
Hippuric acid			

**Biogenic Amines**			
Ac-Orn	Acetylornithine	**Met-SO[1,2,3]**	**Methionine sulfoxide**
ADMA	Asymmetric dimethylarginine	PEA	Phenylethylamine
alpha-AAA	alpha-Aminoadipic acid	**Putrescine[1,2,3]**	**Putrescine**
c4-OH-Pro	cis-4-Hydroxyproline	**Sarcosine[1,2]**	**Sarcosine**
Carnosine[3]	Carnosine	Serotonin	Serotonin
**Creatinine[1,2]**	**Creatinine**	**Spermidine[1,2]**	**Spermidine**
Dopamine	Dopamine	**Spermine[1,2]**	**Spermine**
**Histamine[1,2,3]**	**Histamine**	**t4-OH-Pro[1,2]**	**trans-4-Hydroxyproline**
**Kynurenine[1,2,3]**	**Kynurenine**	**Taurine[1,2]**	**Taurine**
Methylhistidine	Methylhistidine	**total DMA[1,2]**	**Dimethylamine**
**Glycerophospholipids**			
**LysoPC a C14:0[1,2]**	**LysoPhosphatidylcholine acyl C14:0**	LysoPC a C28:1	LysoPhosphatidylcholine acyl C28:1
**LysoPC a C16:1[1,2]**	**LysoPhosphatidylcholine acyl C16:1**	LysoPC a C28:0	LysoPhosphatidylcholine acyl C28:0
**LysoPC a C16:0[1,2]**	**LysoPhosphatidylcholine acyl C16:0**	PC aa C32:2	Phosphatidylcholine diacyl C32:2
**LysoPC a C17:0[1,2]**	**LysoPhosphatidylcholine acyl C17:0**	PC ae C36:0	Phosphatidylcholine acyl-alkyl C36:0
**LysoPC a C18:2[1,2,3]**	**LysoPhosphatidylcholine acyl C18:2**	PC aa C36:6	Phosphatidylcholine diacyl C36:6
**LysoPC a C18:1[1,2]**	**LysoPhosphatidylcholine acyl C18:1**	PC aa C36:0	Phosphatidylcholine diacyl C36:0
**LysoPC a C18:0[1,2]**	**LysoPhosphatidylcholine acyl C18:0**	PC aa C38:6	Phosphatidylcholine diacyl C38:6
LysoPC a C20:4	LysoPhosphatidylcholine acyl C20:4	Pc aa C38:0	Phosphatidylcholine diacyl C38:0
**LysoPC a C20:3[2]**	**LysoPhosphatidylcholine acyl C20:3**	Pc ae C40:6	Phosphatidylcholine acyl-alkyl C40:6
LysoPC a C24:0	LysoPhosphatidylcholine acyl C24:0	PC aa C40:6	Phosphatidylcholine diacyl C40:6
LysoPC a C26:1	LysoPhosphatidylcholine acyl C26:1	PC aa C40:2	Phosphatidylcholine diacyl C40:2
LysoPC a C26:0	LysoPhosphatidylcholine acyl C26:0	PC aa C40:1	Phosphatidylcholine diacyl C40:1
**Lipids and lipid-like molecules**			
Butyric acid			
**Organic acids and derivatives**			
**Betaine[2]**		Citric acid[3]	
**Creatine[2]**		Succinic acid[3]	
Diacetylspermine		**Fumaric acid[1,2]**	
**Lactic acid[1,2,3]**		Pyruvic acid	
**beta-Hydroxybutyric acid[1,2,3]**		Isobutyric acid	
alpha-Ketoglutaric acid		Methylmalonic acid	
DOPA	l-3,4-dihydroxyphenylalanine	Nitro-Tyr	Nitrotyrasine
Propionic acid		p-Hydroxyhippuric acid	para-hydroxyhippuric acid
**Organoheterocyclic compounds**			
Indole acetic acid		**Uric acid[1,2]**	
**Organic nitrogen compounds**			
**Choline[1,2]**	**Choline**	TMAO	Trimethylamine N-oxide
**Hexose**			
Glucose[3]			
**Phenylpropanoids and polyketides**			
HPHPA	3-(3-Hydroxyphenyl)-3-hydroxypropanoic acid		

Sphingomyelins			
SM(OH) C14:1	Hydroxysphingomyeline C14:1	SM C18:0	Sphingomyeline C18:0
SM C16:1	Sphingomyeline C16:1	SM C20:2	Sphingomyeline C20:2
**SM C16:0[1,2.3]**	**Sphingomyeline C16:0**	SM(OH) C22:2	Hydroxysphingomyeline C22:2
SM(OH) C16:1	Hydroxysphingomyeline C16:1	SM(OH) C22:1	Hydroxysphingomyeline C22:1
SM C18:1	Sphingomyeline C18:1	SM(OH) C24:1	Hydroxysphingomyeline C24:1

[1]Analytes significantly different in patient samples as compared with blank VTM (P<0.05)

[2]Analyte concentration in patient samples >2X concentration in blank VTM.

[3]Analytes determined to be significant by feature selection in the Resp or COVID19 models.

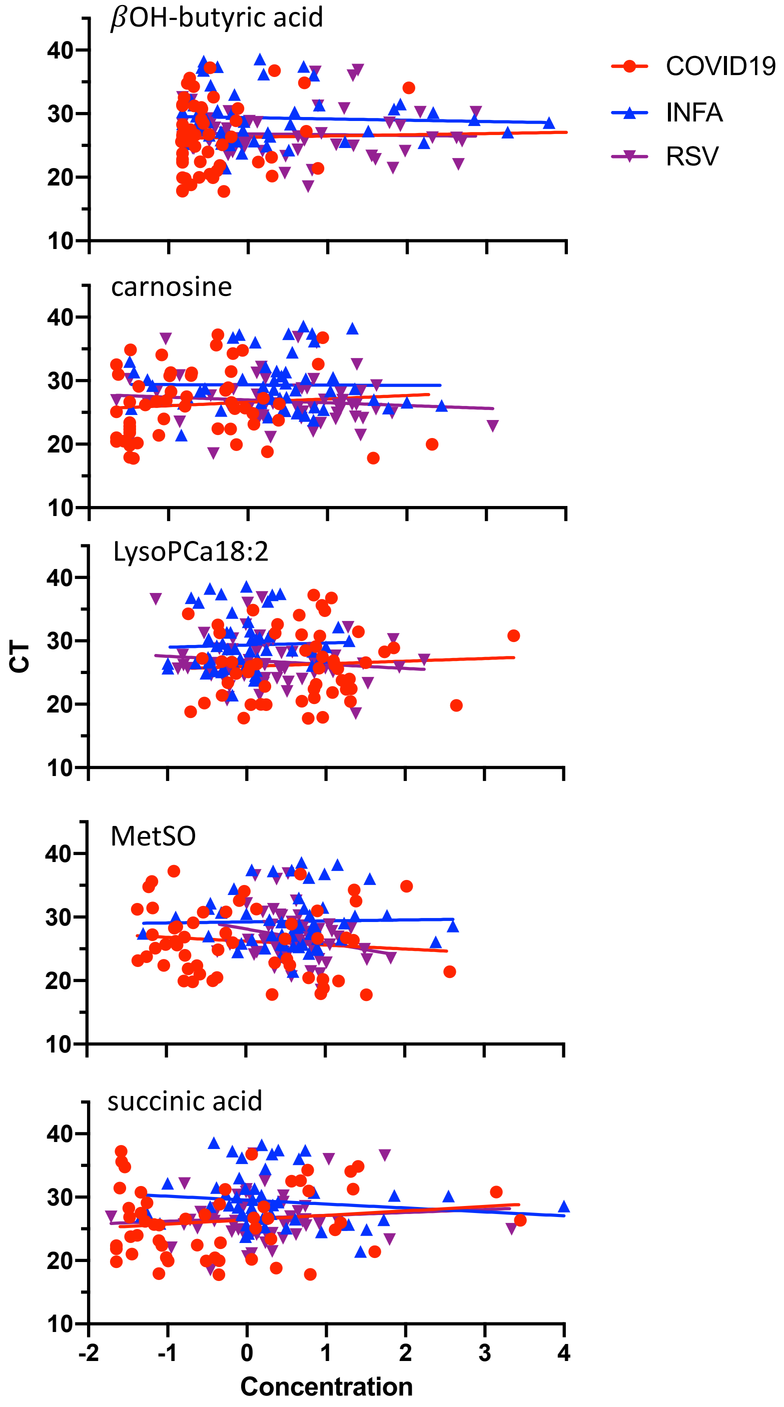

Figure S1: Correlation of cycle threshold (CT) with selected metabolites from the COVID model.

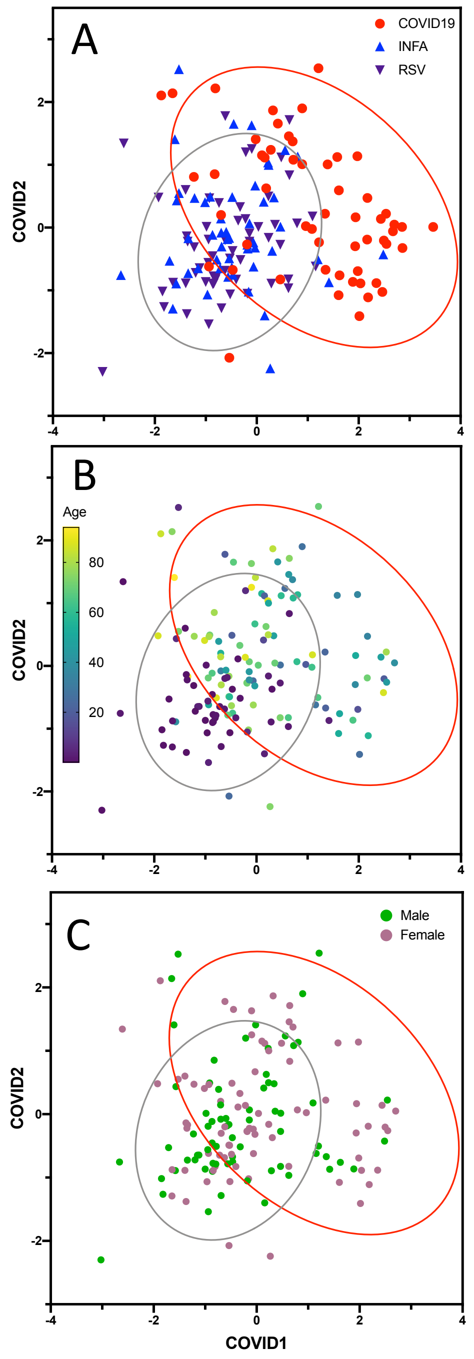

Figure S2: COVID19 model stratified by age and biological sex. OPLS-DA model comparing metabolite profiles in COVID19 patients and patients with INFA or RSV, as presented in Figure 2D (A). Where known, data points are labeled for patient age (B) or biological sex (C). The 95% confidence region is shown for each patient group in the COVID19 model.

Supplementary Table 2: Assay performance parameters for prioritized analytes measured by LC-MS/MS

Analyte ID	QC1[1]				QC2[1]				QC3[1]				VTM[1,4]				
	[Target] µM	[Mean] µM	Diff.[2] %	CV[3] %	[Target] µM	[Mean] µM	Diff.[2] %	CV[3] %	[Target] µM	[Mean] µM	Diff.[2] %	CV[3] %	FC[4]	[Target] µM	[Mean] µM	Diff.[2] %	CV[3] %
Ala	160	203.1	-26.9	11.0	640	764.0	-19.4	10.7	1280	1438.9	-12.4	10.7	28.7	40	54.0	35.0	20.0
Arg	40	46.5	-16.3	4.8	160	176.7	-10.4	6.2	320	355.9	-11.2	4.7	18.1	10	13.5	35.2	8.5
βOH-butyric acid	12.5	13.5	-8.0	5.6	50	51.7	-3.5	5.6	100	104.7	-4.7	4.4	6.8[6]	10	8.6	-14.3	5.4
carnosine	8	9.4	-17.3	9.0	32	35.7	-11.7	7.8	64	71.6	-11.9	5.5	1.2	2	3.2	58.0	7.5
citric acid	12.5	13.4	-6.9	9.3	50	49.5	1.0	5.8	100	99.9	0.1	3	0.4	10	11.6	16.0	3.5
Gly	200	238.6	-19.3	16.5	800	829.9	-3.7	6.1	1600	1647.8	-3.0	10.9	14.0	50	51.6	3.2	33.0
His	40	34.6	13.4	6.8	160	134.1	16.2	6.8	320	265.4	17.0	7.3	25.3	10	13.1	31.2	50.1
histamine	8	8.4	-4.6	14.4	32	33.2	-3.8	11.1	64	65.4	-2.2	14.8	4.9[5]	2	3.4	71.0	15.8
Ile	40	45.0	-12.5	6.2	160	159.2	0.5	8.1	320	306.1	4.3	10.3	26.0	10	13.0	29.8	28.7
kynurenine	40	50.8	-27.1	6.6	160	178.8	-11.7	5.0	320	348.1	-8.8	9.9	7.8[6]	10	8.3	-17.0	10.6
lactic acid	125	137.0	-9.6	7.5	500	517.1	-3.4	5.1	1000	1002.0	-0.2	4.7	4.4	100	115.0	15.0	7.7
Lys	40	51.3	-28.2	11.4	160	183.2	-14.5	8.7	320	328.2	-2.6	9.7	24.3	10	13.6	35.8	14.6
Met	40	44.5	-11.3	9.7	160	173.0	-8.1	4.0	320	335.6	-4.9	7.5	22.8[6]	10	11.9	19.0	7.9
MetSO	8	8.5	-6.8	17.4	32	33.5	-4.8	8.6	64	72.1	-12.7	8.2	28.5[5]	2	2.3	16.0	23.6
Orn	8	9.6	-20.0	9.2	32	33.5	-4.8	10.8	64	62.4	2.5	13.6	8.1	2	2.9	45.0	85.0
Phe	40	48.2	-20.6	8.4	160	184.7	-15.4	6.5	320	342.6	-7.0	5.5	23.8	10	13.3	33.4	24.4
Pro	80	98.3	-22.9	12.8	320	373.6	-16.7	4.9	640	652.4	-1.9	8.0	21.6	20	24.4	22.0	18.2
putrescine	0.8	1.2	-45.1	58.2	3.2	3.7	-17.0	21.6	6.4	6.5	-1.5	11.2	7.7	0	0.3	70.0	29.9
Ser	40	44.3	-10.8	17.7	160	165.2	-3.3	13.7	320	364.1	-13.8	16.1	26.6	10	21.2	112.0	137.7
succinic acid	2.5	2.5	3.8	0.5	10	9.6	3.7	3.1	20	19.3	3.6	1.5	1.5[5]	2	2.3	15.6	12.9
Thr	40	48.8	-22.1	12.1	160	172.1	-7.6	8.3	320	313.4	2.0	9.6	18.7	10	12.5	25.2	58.1
Tyr	40	39.1	2.3	9.9	160	149.7	6.5	4.8	320	274.3	14.3	13.9	9.1	10	8.6	-13.6	24.9
Val	80	93.0	-16.3	9.8	320	350.9	-9.7	6.9	640	702.8	-9.8	7.8	34.7	20	24.6	23.0	27.9

[1] Quality control material based on solution standards (QC1-3), or spiked viral transport medium (VTM). Each QC sample was assayed 9 times over 3 days.
[2] % difference of mean measured concentration relative to target.
[3] Total coefficients of variation (CV) for spiked VTM, based on 9 measurements over 3 days.
[4] Fold-change of mean concentration in blank VTM (N=12) as compared with mean concentration in patient samples. All other parameters for VTM are based on spiked VTM samples. All %CVs for analyte measurements in blank VTM were <30, with the exception of the analytes noted below.
[5] CVs of 30-40%. Concentration of MetSO and histamine in blank VTM were below the lowest calibrator and estimated lower limit of quantification of 0.3uM.
[6] CVs of >40%. Concentration of Met, kynurenine and βOH-butyric acid in blank VTM were below the lowest calibrator and estimated lower limit of quantification of 1.7uM.

Supplementary Methods

NOTE: Raw data and fully reproducible code for this project are available on GitHub: http://bit.ly/COVID-Metabolomics

Setup

Basic setup for plotting and data handling

```
library(tidyverse) # Tools for data science (graphing, data reorganizing, etc
.)
library(ropls)
library(pROC)

# Some custom graphing stuff
source("./theme_pub.R")
theme_set(theme_pub())
```

User-parameters

```
flipResp<-T # If True, reverse main axis scaling for respiratory model
flipCOVID<-T # If True, reverse main axis scaling for COVID model
```

Load data

```
featDatA<-read.csv("./data/FeatDatA.csv") # Features selected from resDat usi
ng Subset A
featDatB<-read.csv("./data/FeatDatB.csv") # Features selected from resDat usi
ng Subset B
featDatC<-read.csv("./data/FeatDatC.csv") # Features selected from resDat usi
ng Subset C
featDatCT<-read.csv("./data/FeatDatCT.csv") # Features selected from resDat u
sing Subset based on correlation with CT
```

Pipeline Details

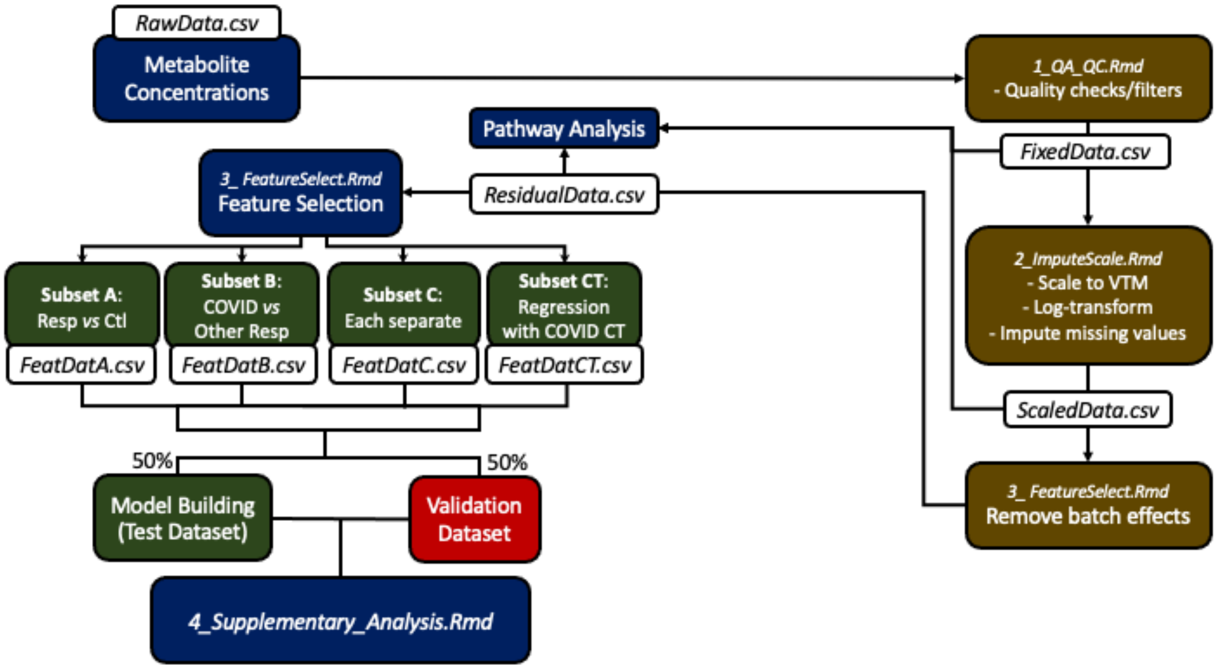

Analysis Pipeline

PLS-DA - all groups

Full Partial Least Squares Discriminant Analysis with all 4 groups (and 3 orthogonal predictor axes)

NOTE: This is used for graphing purposes only. For predictive models, see OPLS-DA models, below.

```
# RDA Model

# Setup for grid search with Leave-One-Out Cross-Validation (LOOC using the t
est-building subset of data)

FULLdat<-featDatC %>% # Dataset with new encoding

  filter(Class.name %in% c("Control","COVID19","Influenza","RSV")) %>% # Remo
ve VTM

  column_to_rownames("Sample.Name")


DescNames<-c("Batch.Number","Class.name","Sex","Age","CT","OrigClass") # Resp
onse Variable

Concs<-names(FULLdat)[!names(FULLdat) %in% DescNames] # Predictor Variables
```

```
# Organize data for opls
metData<-FULLdat[,Concs] # Metabolite data
patClass<-FULLdat[,"Class.name"] # Predictors
# Set row.names
names(patClass)<-row.names(FULLdat)

# Model of full data for plotting
FULLmod<-opls(metData, patClass, predI=3, fig.pdfC="none")
```

```
## PLS-DA
## 210 samples x 31 variables and 1 response
## standard scaling of predictors and response(s)
##       R2X(cum) R2Y(cum) Q2(cum) RMSEE pre ort pR2Y  pQ2
## Total    0.663    0.395   0.334 0.343   3   0 0.05 0.05
```

NOTE: No confusion matrix is calculated here (no cross-validation). The purpose is to see whether samples form distinct groups, and factor loadings, rather than to generate and test predictions from the model (that is done below).

PLS-DA axis plots

```
pDatF<-as.data.frame(FULLmod@scoreMN)
pDatF$Class<-as.factor(FULLdat$Class.name)
pDatF$Age<-as.factor(FULLdat$Age)
pDatF$Sex<-as.factor(FULLdat$Sex)

ggplot(aes(x=p1,y=p2,group=Class,fill=Class,shape=Class),data=pDatF) +
  stat_ellipse(aes(colour=Class),size=1.2, alpha=0.8) +
  geom_point(size=3,alpha=0.8) +
  scale_fill_manual(values=c("#989788","#E54F6D","#008BF8","#623CEA","#E7EBC5
")) +
  scale_colour_manual(values=c("#989788","#E54F6D","#008BF8","#623CEA")) +
  scale_shape_manual(values=c(22,21,24,25,22))
```

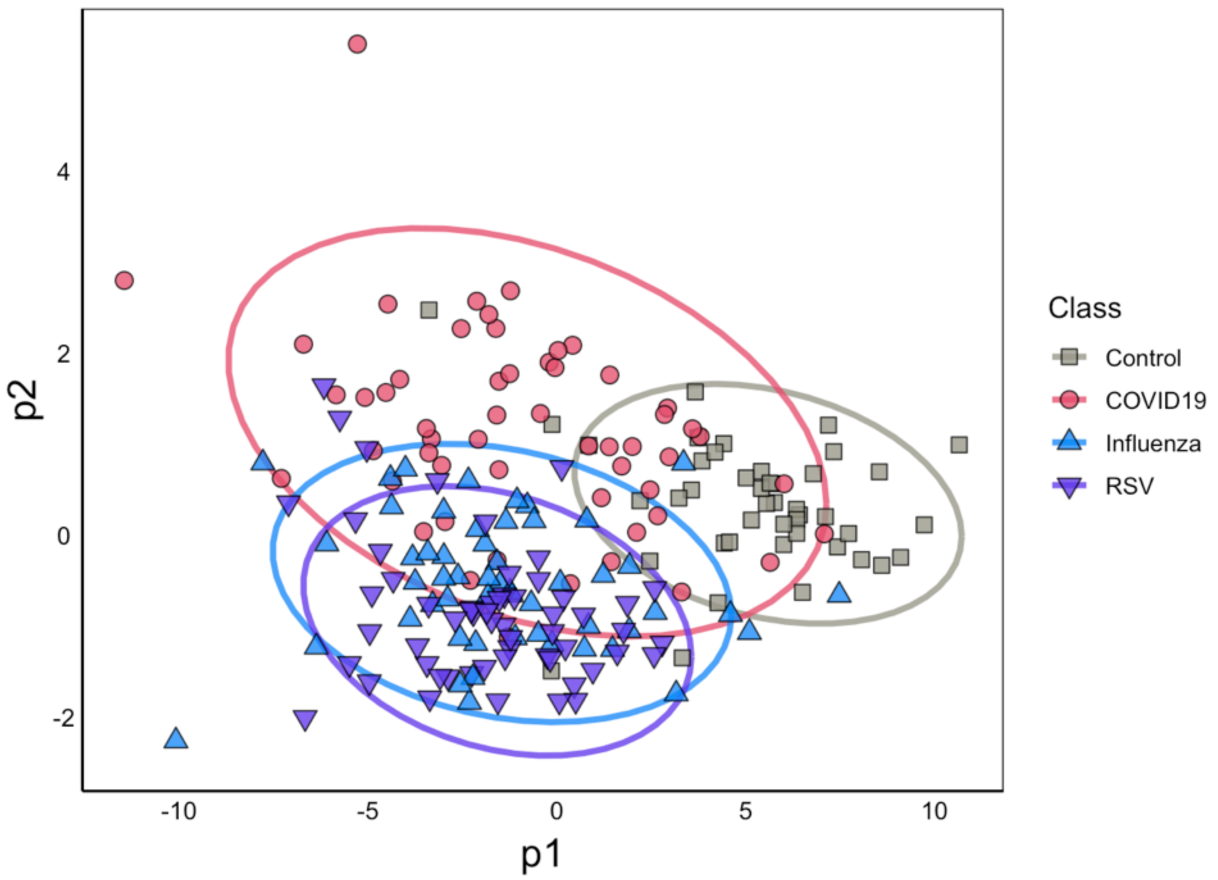


```
ggplot(aes(x=p1,y=p3,group=Class,fill=Class,shape=Class),data=pDatF) +
  stat_ellipse(aes(colour=Class),size=1.2, alpha=0.8) +
  geom_point(size=3,alpha=0.8) +
  scale_fill_manual(values=c("#989788","#E54F6D","#008BF8","#623CEA","#E7EBC5
")) +
  scale_colour_manual(values=c("#989788","#E54F6D","#008BF8","#623CEA")) +
  scale_shape_manual(values=c(22,21,24,25,22))
```

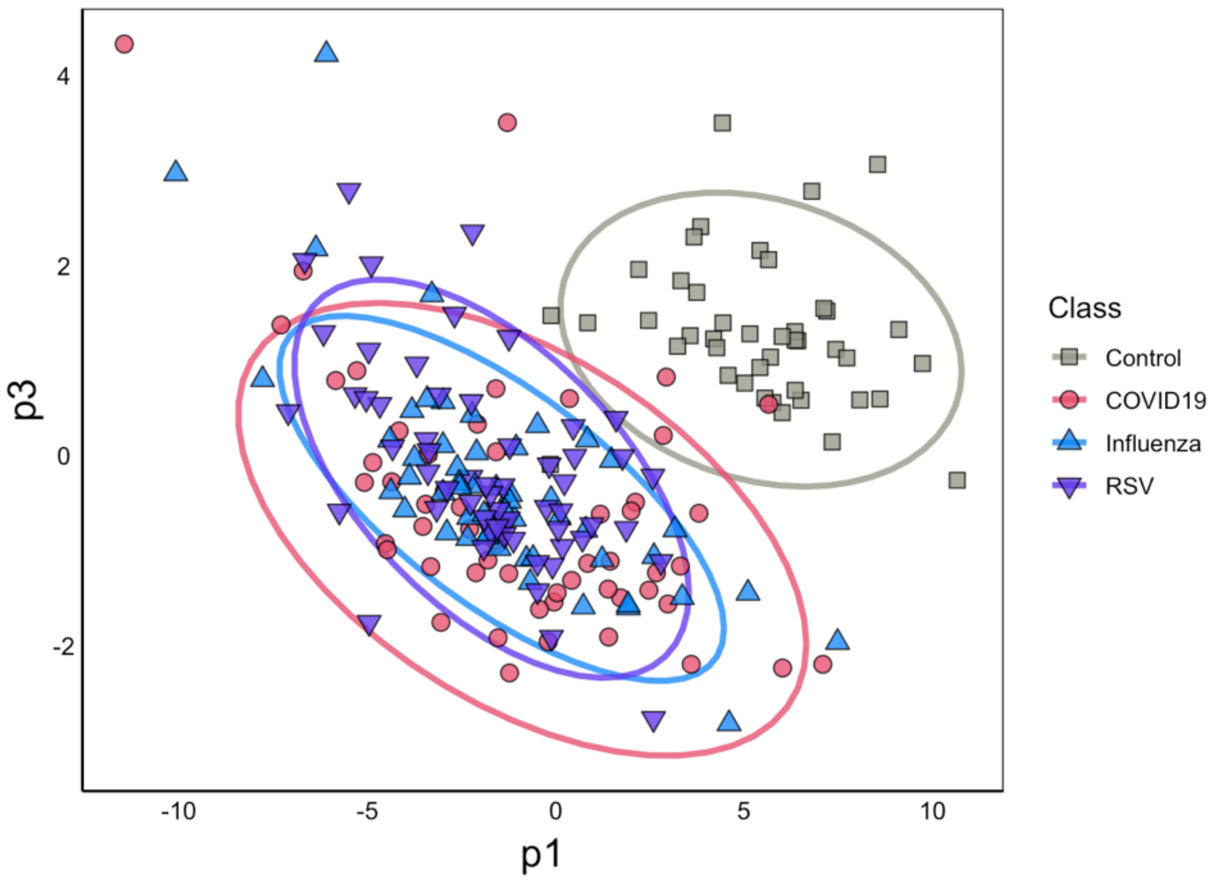


```
ggplot(aes(x=p2,y=p3,group=Class,fill=Class,shape=Class),data=pDatF) +
  stat_ellipse(aes(colour=Class),size=1.2, alpha=0.8) +
  geom_point(size=3,alpha=0.8) +
  scale_fill_manual(values=c("#989788","#E54F6D","#008BF8","#623CEA","#E7EBC5
")) +
  scale_colour_manual(values=c("#989788","#E54F6D","#008BF8","#623CEA")) +
  scale_shape_manual(values=c(22,21,24,25,22))
```

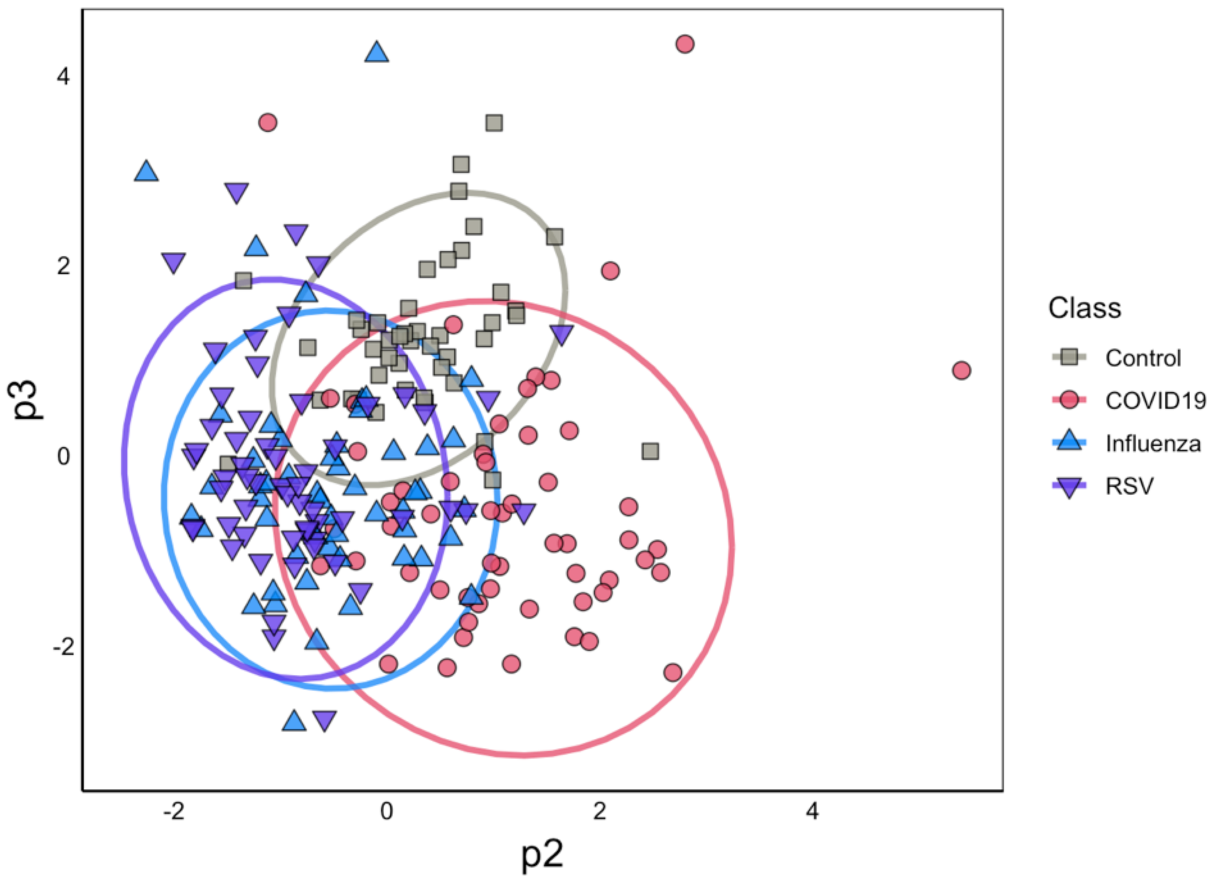

Export PLD-DA plotting data

```
#write.csv(pDatF,"./pDat/FULLdat.csv")
```

Graph of loadings:

```
Loadings<-as.data.frame(FULLmod@loadingMN)
Loadings$Metabolite<-row.names(Loadings)
heatDat<-gather(Loadings,Axis,Loading,all_of(names(Loadings[-4])))
heatDat<-as.data.frame(heatDat)

ggplot(aes(x=Axis,y=Metabolite,fill=Loading),data=heatDat) + geom_tile() +
  facet_grid(~ Axis, scales = "free_x", space = "free_x") +
  scale_fill_gradientn(colours=c("#008BF8","#E7EBC5","#E54F6D"))
```

Export FULL model loadings data

```
#write.csv(heatDat,"./pDat/FULLload.csv")
```

OPLS-DA with FS

Orthogonal PLS used here for models based on two bins. NOTE: the x-axis is the orthogonal predictor, a second (y-axis) is added only for plotting purposes.

Control vs All respiratory

```
# Respiratory Only
RESPdat<-featDataA %>% # Dataset with new encoding
  filter(Class.name %in% c("Control","COVID19","Influenza","RSV")) %>%
  column_to_rownames("Sample.Name")
RESPdat$OrigClass<-RESPdat$Class.name
RESPdat$Class.name<-recode_factor(RESPdat$Class.name, COVID19 = "Resp",
                                 Influenza = "Resp", RSV = "Resp")

DescNames<-c("Batch.Number","Class.name","Sex","Age","CT","OrigClass") # Resp
onse Variable
Concs<-names(RESPdat)[!names(RESPdat) %in% DescNames] # Predictor Variables

# Organize data for opls
metData<-RESPdat[,Concs] # Metabolite data
patClass<-RESPdat[,"Class.name"] # Predictors
# Set row.names
names(patClass)<-row.names(RESPdat)
# opls model
set.seed(4325)
OPLSMod<-opls(metData, patClass, subset="odd", fig.pdfC="none")
```

```
## Warning: 'permI' set to 0 because train/test partition is selected
## PLS-DA
## 105 samples x 28 variables and 1 response
## standard scaling of predictors and response(s)
```

```
##       R2X(cum) R2Y(cum) Q2(cum) RMSEE RMSEP pre ort
## Total    0.749    0.828   0.787 0.172 0.231   3   0
trainSet <- getSubsetVi(OPLSMod)

print("Fitted Model")
## [1] "Fitted Model"
table(patClass[trainSet],fitted(OPLSMod))
##
##            Resp Control
##   Resp       82       1
##   Control     0      22
print("Test Data")
## [1] "Test Data"
TestFit<-table(patClass[-trainSet],
      predict(OPLSMod, metData[-trainSet, ]))

TestFit
##
##            Resp Control
##   Resp       82       1
##   Control     3      19
TP<-TestFit[1] # True Positive
FP<-sum(TestFit[2])# False Positive
FN<-sum(TestFit[3]) # False Negative
TN<-sum(TestFit)-TP-FP-FN# True Negative

# Model of full data for plotting
pOPLSMod<-opls(metData, patClass, fig.pdfC="none")
## PLS-DA
## 210 samples x 28 variables and 1 response
## standard scaling of predictors and response(s)
##       R2X(cum) R2Y(cum) Q2(cum) RMSEE pre ort pR2Y  pQ2
## Total    0.722    0.771    0.72 0.197   3   0 0.05 0.05
```

ROC

```
ROCrespTrain<-roc(patClass[trainSet],pOPLSMod@scoreMN[trainSet,1])
## Setting levels: control = Resp, case = Control
## Setting direction: controls < cases
ROCrespTest<-roc(patClass[-trainSet],pOPLSMod@scoreMN[-trainSet,1])
## Setting levels: control = Resp, case = Control
## Setting direction: controls < cases
ggroc(data=ROCrespTrain,colour="blue") + ggtitle("Training Data") +
  xlab("Specificity") + ylab("Sensitivity") + xlim(1,0) +
  geom_abline(intercept=1,slope=1,colour="grey")
## Scale for 'x' is already present. Adding another scale for 'x', which will
## replace the existing scale.
```

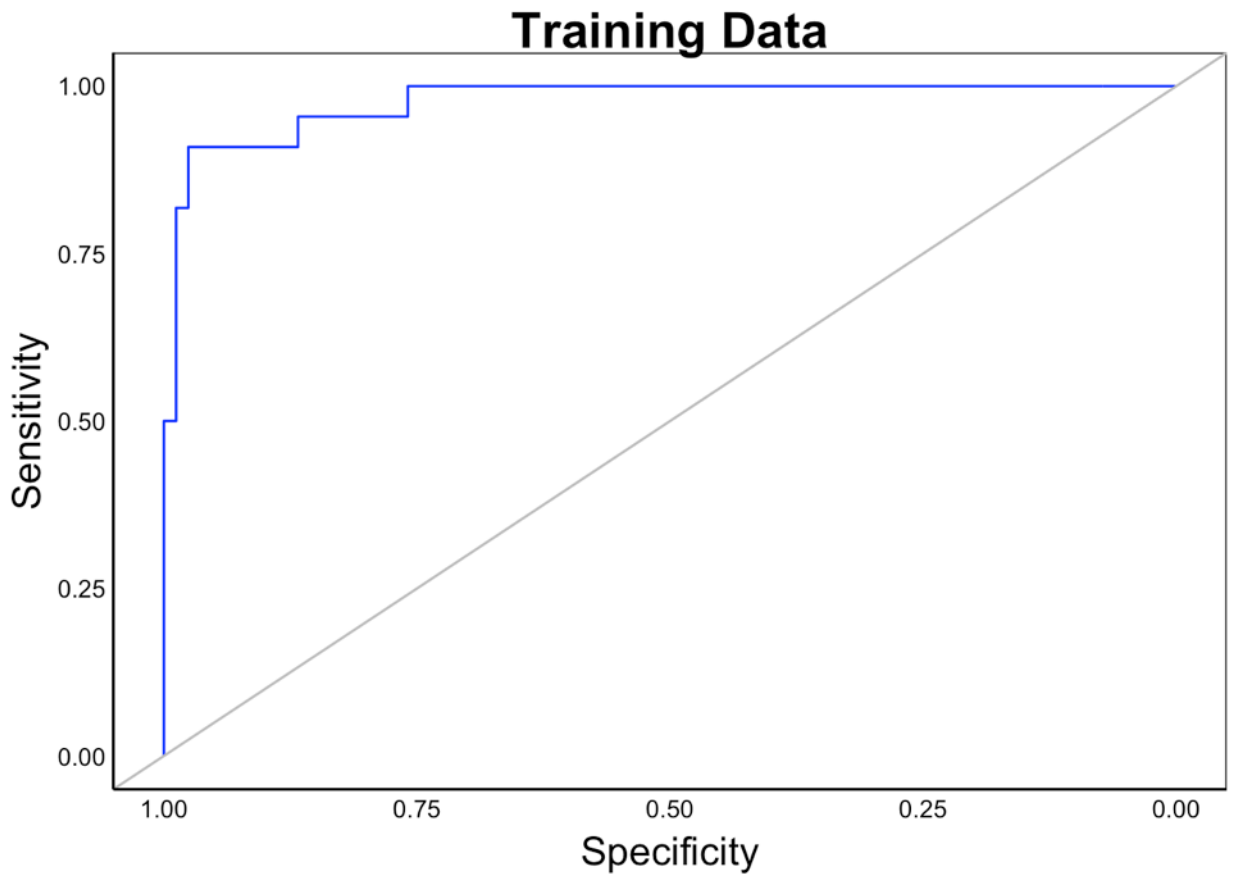

```r
ggroc(data=ROCrespTest,colour="red") + ggtitle("Test Data") +
  xlab("Specificity") + ylab("Sensitivity") + xlim(1,0) +
  geom_abline(intercept=1,slope=1,colour="grey")
## Scale for 'x' is already present. Adding another scale for 'x', which will
## replace the existing scale.
```

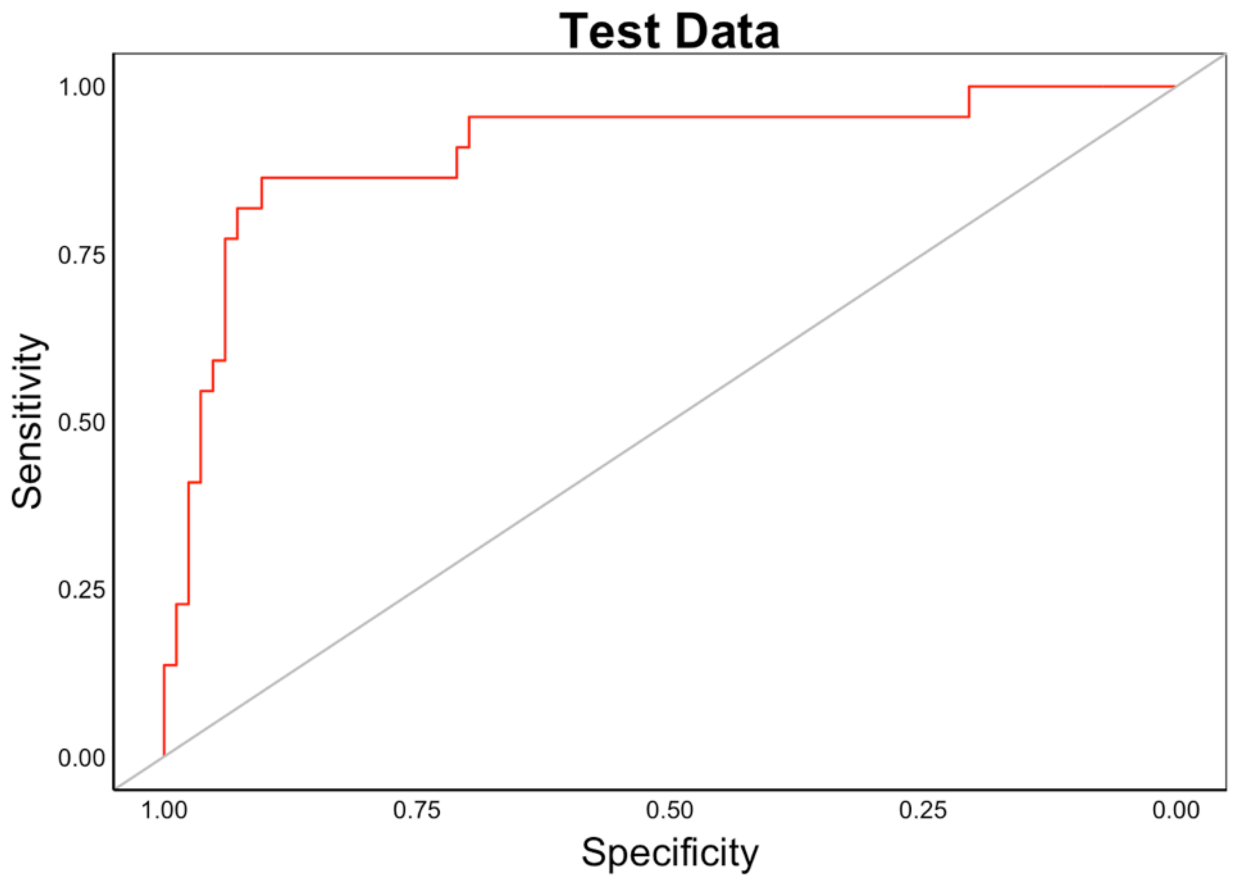

```r
print("Training Data AUC")
## [1] "Training Data AUC"
print(ROCrespTrain)
##
## Call:
## roc.default(response = patClass[trainSet], predictor = pOPLSMod@scoreMN[tr
ainSet,      1])
##
## Data: pOPLSMod@scoreMN[trainSet, 1] in 83 controls (patClass[trainSet] Res
p) < 22 cases (patClass[trainSet] Control).
## Area under the curve: 0.977
print("------------------------------")
## [1] "------------------------------"
print("Validation Data AUC")
## [1] "Validation Data AUC"
print(ROCrespTest)
##
## Call:
## roc.default(response = patClass[-trainSet], predictor = pOPLSMod@scoreMN[-
trainSet,      1])
##
## Data: pOPLSMod@scoreMN[-trainSet, 1] in 83 controls (patClass[-trainSet] R
esp) < 22 cases (patClass[-trainSet] Control).
## Area under the curve: 0.9058
```

Accuracy

```r
(TP+TN)/(sum(TestFit))
## [1] 0.9619048
```

Sensitivity

```r
(TP)/(TP+FN)
## [1] 0.9879518
```

Specificity

```
TN/(TN+FP)
## [1] 0.8636364
```

Plot data

NOTE: plot for training data only

```
pDat<-as.data.frame(pOPLSMod@scoreMN)
if(flipResp==T){
  pDat<-pDat*-1
}
pDat$Class<-RESPdat$OrigClass
pDat$Group<-RESPdat$Class.name
pDat$Age<-RESPdat$Age
pDat$Sex<-RESPdat$Sex
names(pDat)<-gsub("p([0-9])","Resp\\1",names(pDat))

#ggplot(aes(x=Full1,y=Full2,group=Class),data=pDat) +
#  geom_point(aes(colour=Class),size=3,alpha=0.7) + scale_colour_brewer(palet
te = "Set1")

#ggplot(aes(x=Full3,y=Full4,group=Class),data=pDat) +
#  geom_point(aes(colour=Class),size=3,alpha=0.7) + scale_colour_brewer(palet
te = "Set1")

#ggplot(aes(x=Full1,y=Full5,group=Class),data=pDat) +
#  geom_point(aes(colour=Class),size=3,alpha=0.7) + scale_colour_brewer(palet
te = "Set1")
```

Plot Control vs All Resp

```
ggplot(aes(x=Resp1,y=Resp2),data=pDat) +
  stat_ellipse(aes(colour=Group),size=1.2, alpha=0.8) +
  geom_point(aes(fill=Class,shape=Class),size=3,alpha=0.8) +
```

```
  scale_fill_manual(values=c("#989788","#E54F6D","#008BF8","#623CEA","#E7EBC5
")) +
  scale_colour_manual(values=c("grey65","#E54F6D")) +
  scale_shape_manual(values=c(22,21,24,25,22))
```

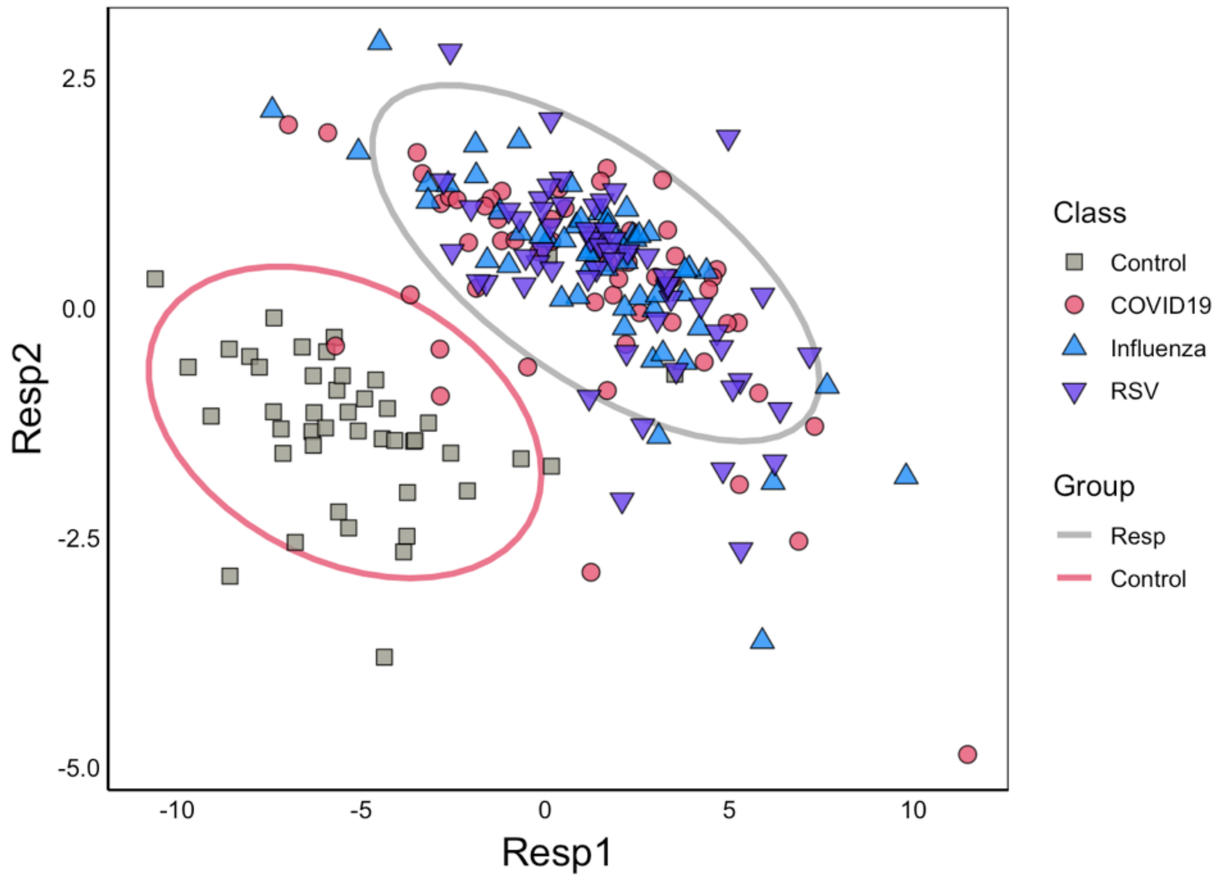

And the same showing age & sex

```
ggplot(aes(x=Resp1,y=Resp2,group=Class),data=pDat) +
geom_point(aes(colour=as.numeric(Age),shape=Sex),size=3,alpha=0.8) +
  scale_colour_gradient(low="blue",high="pink")
## Warning in FUN(X[[i]], ...): NAs introduced by coercion
## Warning: Removed 16 rows containing missing values (geom_point).
```

Export OPLS data

```
#write.csv(pDat,"./pDat/RESPdat.csv")
```

COVID vs Other respiratory

```
# Respiratory Only
COVIDdat<-featDatB %>% # Dataset with new encoding
    filter(Class.name %in% c("COVID19","Influenza","RSV")) %>%
    column_to_rownames("Sample.Name")
COVIDdat$OrigClass<-COVIDdat$Class.name
COVIDdat$Class.name<-gsub("Influenza|RSV","Other Resp",COVIDdat$Class.name)

DescNames<-c("Batch.Number","Class.name","Sex","Age","CT","OrigClass") # Resp
onse Variable
Concs<-names(COVIDdat)[!names(COVIDdat) %in% DescNames] # Predictor Variables

# Organize data for opls
metData<-COVIDdat[,Concs] # Metabolite data
patClass<-COVIDdat[,"Class.name"] # Predictors
# Set row.names
names(patClass)<-row.names(COVIDdat)
# opls model
OPLSMod2<-opls(metData, patClass, predI = 2, subset="odd", fig.pdfC="none")
```

```
## Warning: 'permI' set to 0 because train/test partition is selected
## PLS-DA
## 84 samples x 5 variables and 1 response
## standard scaling of predictors and response(s)
##       R2X(cum) R2Y(cum) Q2(cum) RMSEE RMSEP pre ort
## Total    0.536    0.442   0.301 0.359  0.38   2   0
```

```
trainSet <- getSubsetVi(OPLSMod2)

print("Fitted Model")
```

```
## [1] "Fitted Model"
```

```
table(patClass[trainSet],fitted(OPLSMod2))
##
##              COVID19 Other Resp
##   COVID19         21          7
##   Other Resp       6         50
print("Test Data")
## [1] "Test Data"
TestFit<-table(patClass[-trainSet],
      predict(OPLSMod2, metData[-trainSet, ]))

TestFit
##
##              COVID19 Other Resp
##   COVID19         20          7
##   Other Resp       5         50
TP<-TestFit[1] # True Positive
FP<-sum(TestFit[2])# False Positive
FN<-sum(TestFit[3]) # False Negative
TN<-sum(TestFit)-TP-FP-FN# True Negative

# Model for plotting full dataset
pOPLSMod2<-opls(metData, patClass,fig.pdfC="none")
## PLS-DA
## 166 samples x 5 variables and 1 response
## standard scaling of predictors and response(s)
##       R2X(cum) R2Y(cum) Q2(cum) RMSEE pre ort pR2Y  pQ2
## Total    0.566    0.404   0.341 0.367   2   0 0.05 0.05
```

ROC

```
ROCcovidTrain<-roc(patClass[trainSet],pOPLSMod2@scoreMN[trainSet,1])
## Setting levels: control = COVID19, case = Other Resp
## Setting direction: controls < cases
ROCcovidTest<-roc(patClass[-trainSet],pOPLSMod2@scoreMN[-trainSet,1])
## Setting levels: control = COVID19, case = Other Resp
```

```
## Setting direction: controls < cases
ggroc(data=ROCcovidTrain,colour="blue") + ggtitle("Training Data") +
  xlab("Specificity") + ylab("Sensitivity") + xlim(1,0) +
  geom_abline(intercept=1,slope=1,colour="grey")
## Scale for 'x' is already present. Adding another scale for 'x', which will
## replace the existing scale.
```

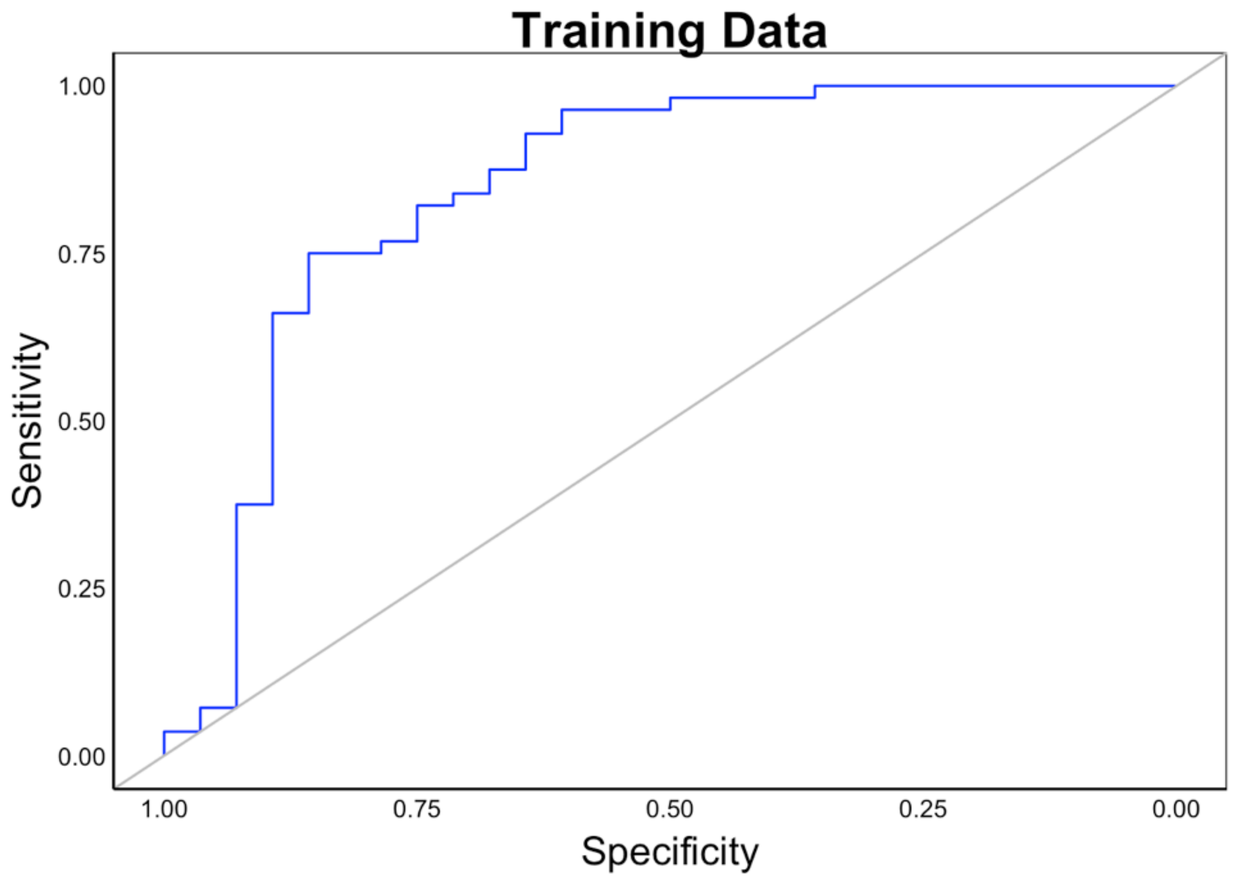


```
ggroc(data=ROCcovidTest,colour="red") + ggtitle("Test Data") +
  xlab("Specificity") + ylab("Sensitivity") + xlim(1,0) +
  geom_abline(intercept=1,slope=1,colour="grey")
## Scale for 'x' is already present. Adding another scale for 'x', which will
## replace the existing scale.
```

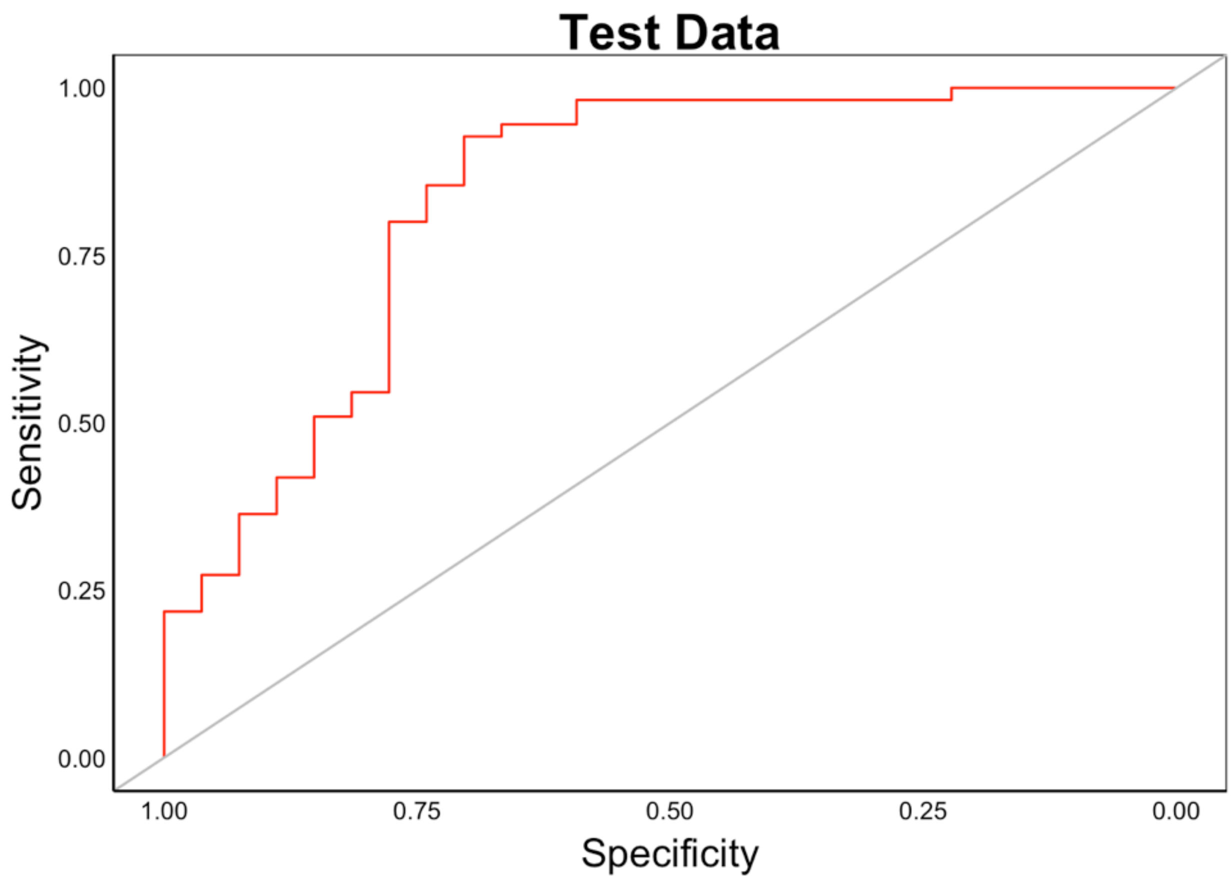


```
print("Training Data AUC")
## [1] "Training Data AUC"
print(ROCcovidTrain)
##
## Call:
## roc.default(response = patClass[trainSet], predictor = pOPLSMod2@scoreMN[t
rainSet,     1])
##
## Data: pOPLSMod2@scoreMN[trainSet, 1] in 28 controls (patClass[trainSet] CO
VID19) < 56 cases (patClass[trainSet] Other Resp).
## Area under the curve: 0.8463
print("-------------------------------")
## [1] "-------------------------------"
print("Validation Data AUC")
## [1] "Validation Data AUC"
```

```
print(ROCcovidTest)
```

```
##
## Call:
## roc.default(response = patClass[-trainSet], predictor = pOPLSMod2@scoreMN[-trainSet,     1])
##
## Data: pOPLSMod2@scoreMN[-trainSet, 1] in 27 controls (patClass[-trainSet] COVID19) < 55 cases (patClass[-trainSet] Other Resp).
## Area under the curve: 0.8377
```

Accuracy

```
(TP+TN)/(sum(TestFit))
```

```
## [1] 0.8536585
```

Sensitivity

```
(TP)/(TP+FN)
```

```
## [1] 0.7407407
```

Specificity

```
TN/(TN+FP)
```

```
## [1] 0.9090909
```

Plot COVID vs other Respiratory

NOTE: plot for training data only

```
pDat2<-as.data.frame(pOPLSMod2@scoreMN)
if(flipCOVID==T){
  pDat2<-pDat2*-1
}
pDat2$Class<-COVIDdat$OrigClass
pDat2$Group<-COVIDdat$Class.name
pDat2$Age<-COVIDdat$Age
pDat2$Sex<-COVIDdat$Sex
```

```
names(pDat2)<-gsub("p([0-9])","COVID\\1",names(pDat2))


ggplot(aes(x=COVID1,y=COVID2),data=pDat2) +
  stat_ellipse(aes(colour=Group),size=1.2, alpha=0.8) +
  geom_point(aes(fill=Class,shape=Class),size=3,alpha=0.8) +
  scale_fill_manual(values=c("#E54F6D","#008BF8","#623CEA")) +
  scale_colour_manual(values=c("#E54F6D","grey65")) +
  scale_shape_manual(values=c(21,24,25))
```

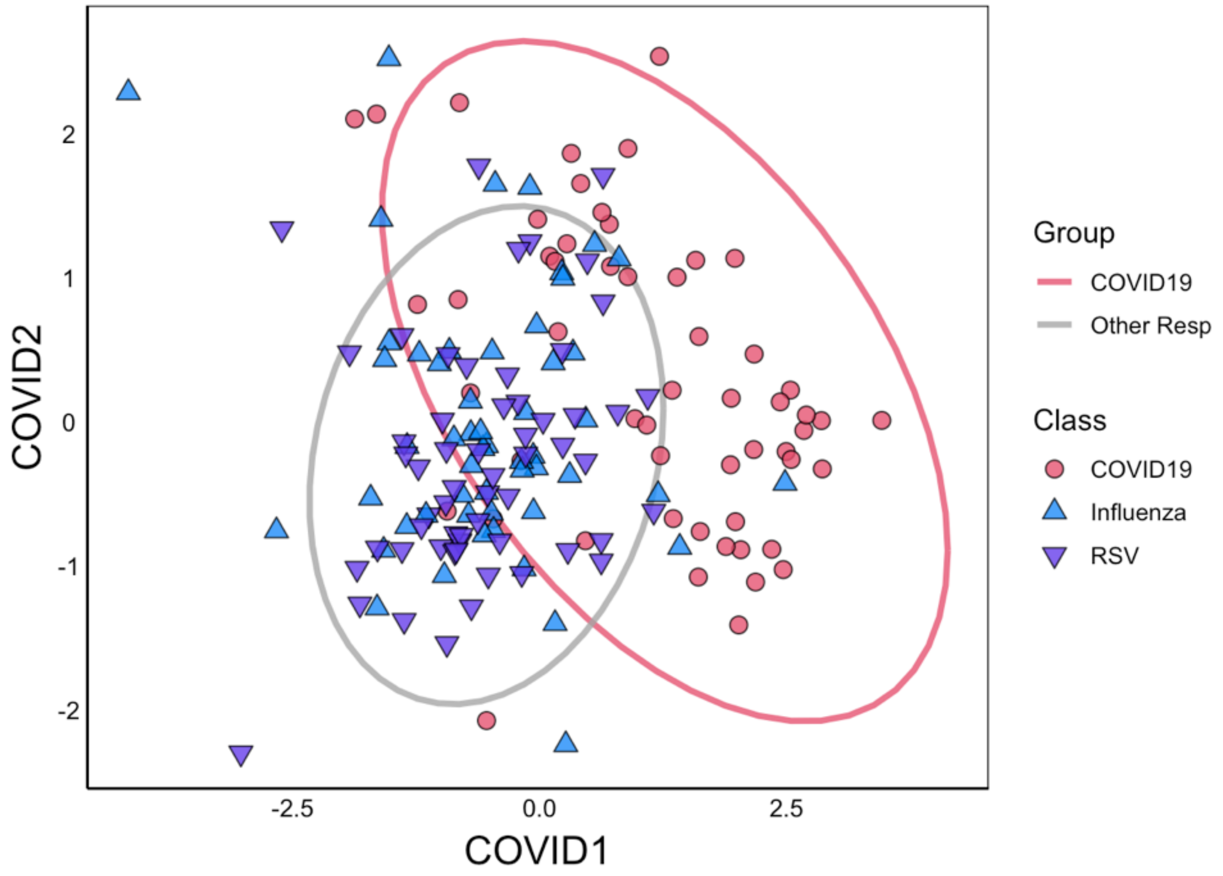

Age & sex

```
ggplot(aes(x=COVID1,y=COVID2,group=Class),data=pDat2) +
geom_point(aes(colour=as.numeric(Age),shape=Sex),size=3,alpha=0.8) +
  scale_colour_gradient(low="blue",high="pink")
## Warning in FUN(X[[i]], ...): NAs introduced by coercion
## Warning: Removed 16 rows containing missing values (geom_point).
```

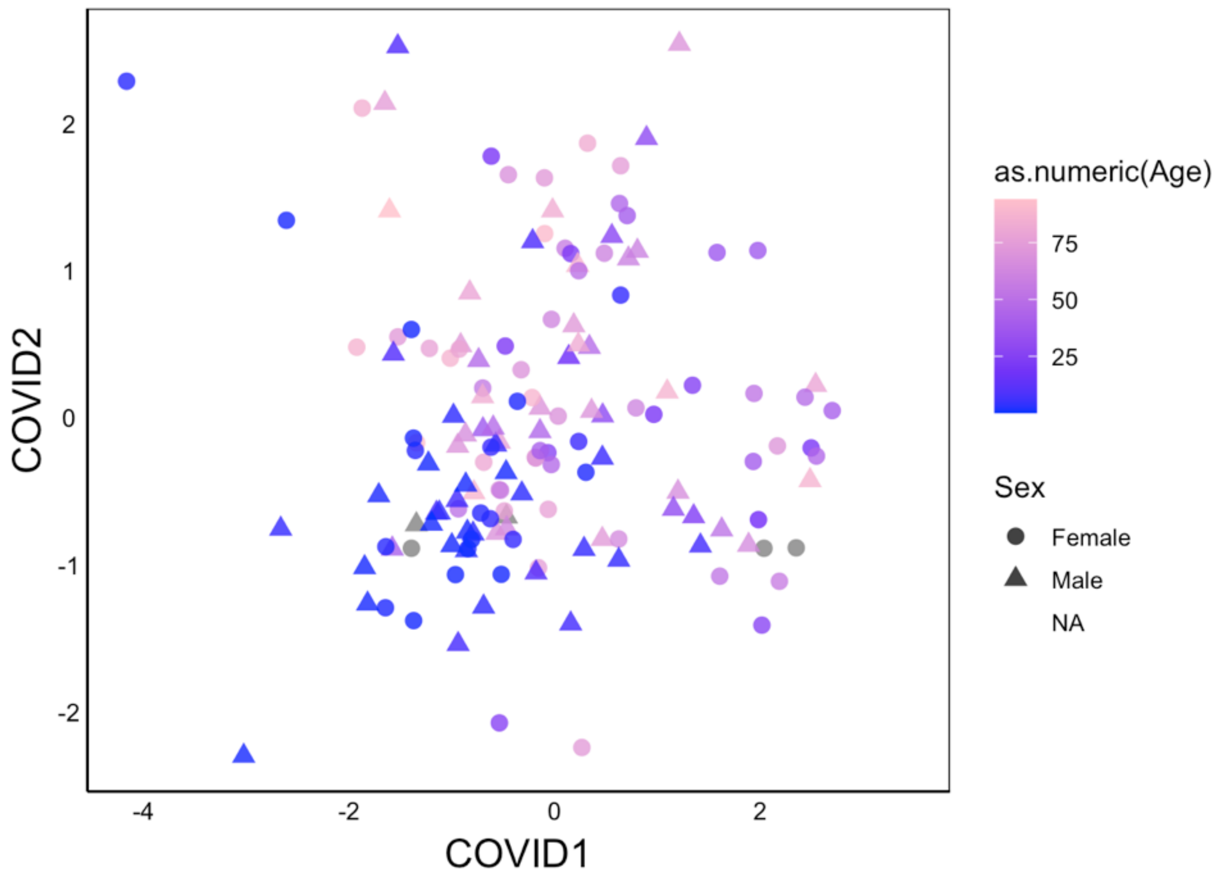

Export OPLS data

```
#write.csv(pDat2,"./pDat/COVIDdat.csv")
```

Loadings

Loading for both OPLS models

RESP = Control vs all respiratory COVID = COVID vs other respiratory

```
Loadings<-as.data.frame(OPLSMod@loadingMN)
names(Loadings)<-gsub("p","Resp",names(Loadings))
if(flipResp==T){
  Loadings<-Loadings*-1
}
cLoadings<-as.data.frame(OPLSMod2@loadingMN)
names(cLoadings)<-gsub("p","COVID",names(cLoadings))
```

```
if(flipCOVID==T){
  cLoadings<-cLoadings*-1
}
heatDat<-full_join(rownames_to_column(Loadings), rownames_to_column(cLoadings
), by = "rowname")
heatDat<-gather(heatDat,Axis,Loading,all_of(names(heatDat)[-1]))
names(heatDat)[1]<-"Metabolite"
heatDat<-as.data.frame(heatDat[heatDat$Axis %in%
                                    c("COVID1","Resp1"), ])


ggplot(aes(x=Axis,y=Metabolite,fill=Loading),data=heatDat) + geom_tile() +
  facet_grid(~ Axis, scales = "free_x", space = "free_x") +
  scale_fill_gradientn(colours=c("#008BF8","#E7EBC5","#E54F6D"))
```

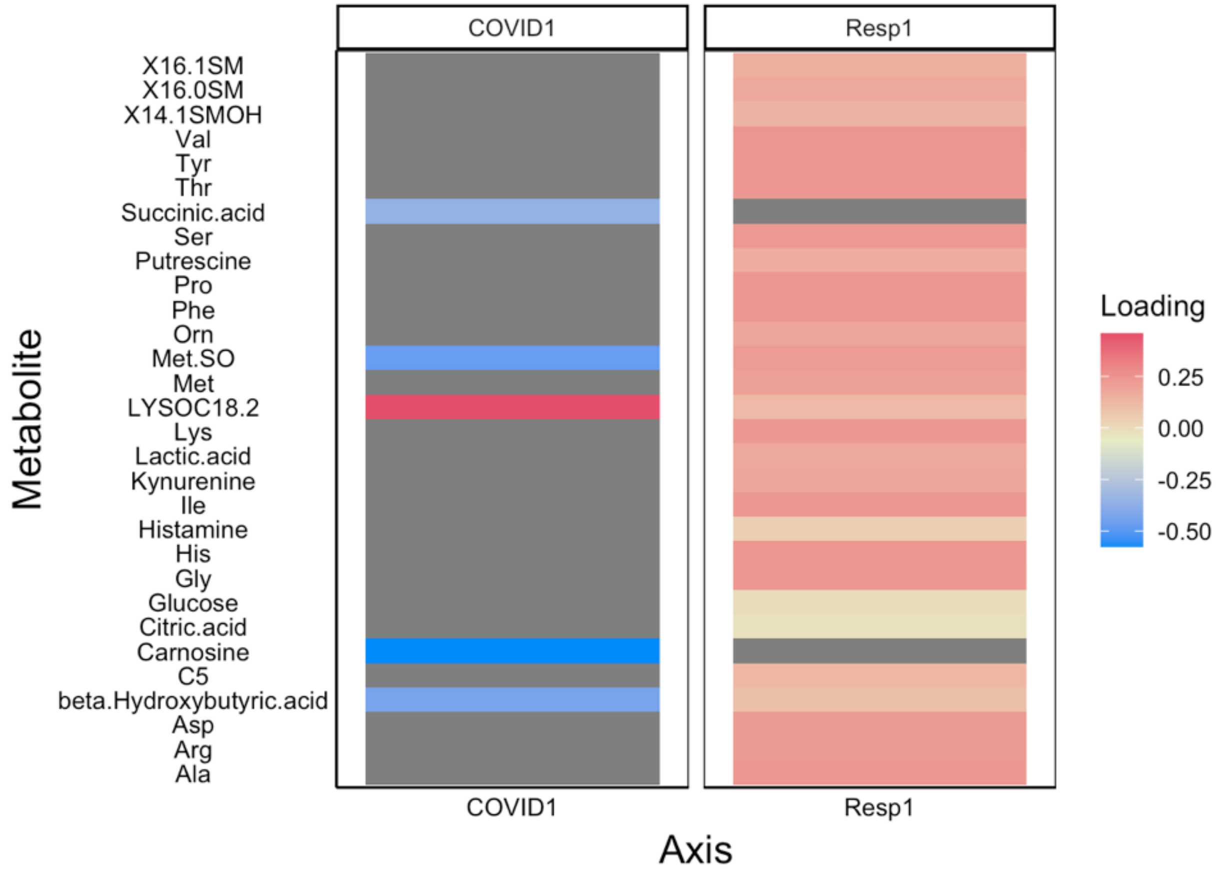

Export OPLS data

```
#write.csv(heatDat,"./pDat/OPLSload.csv")
```

Histogram of significant metabolites

```
pDat3<-gather(FULLdat,Metabolite,Concentration,
              all_of(c("Succinic.acid","Met.SO","LYSOC18.2","Carnosine","beta
.Hydroxybutyric.acid")))
ggplot(aes(x=Concentration,group=Class.name),data=pDat3) +
  geom_density(aes(fill=Class.name),alpha=0.3) + facet_grid(Metabolite ~ .) +
  scale_fill_manual(values=c("#989788","#E54F6D","#008BF8","#623CEA","#E7EBC5
"))
```

Export Metabolite Data

```
#write.csv(FULLdat,"./pDat/Metabolites.csv")
```

Other stuff (Exploratory)

CT Correlations

Do the major metabolites from COVID1 correlate with CT value in COVID and other respiratory patients?

```
COVIDmet<-c("Succinic.acid",
           "Carnosine","Met.SO","LYSOC18.2","beta.Hydroxybutyric.acid")
CTdat<-COVIDdat[,c("OrigClass","Sex","Age","CT",COVIDmet)] %>%
  gather(Metab,Conc,all_of(COVIDmet))

ggplot(aes(x=Conc,y=as.numeric(CT)),data=CTdat) +
  geom_smooth(aes(group=OrigClass,colour=OrigClass),se=F,method="lm") +
  geom_point(aes(group=OrigClass,fill=OrigClass,shape=OrigClass),
             size=3,alpha=0.8) +
```

```
  scale_fill_manual(values=c("#E54F6D","#008BF8","#623CEA")) +
  scale_colour_manual(values=c("#E54F6D","#008BF8","#623CEA")) +
  scale_shape_manual(values=c(21,24,24)) +
  facet_grid(Metab~.,scales="free")
```

```
## `geom_smooth()` using formula 'y ~ x'
## Warning: Removed 25 rows containing non-finite values (stat_smooth).
## Warning: Removed 25 rows containing missing values (geom_point).
```

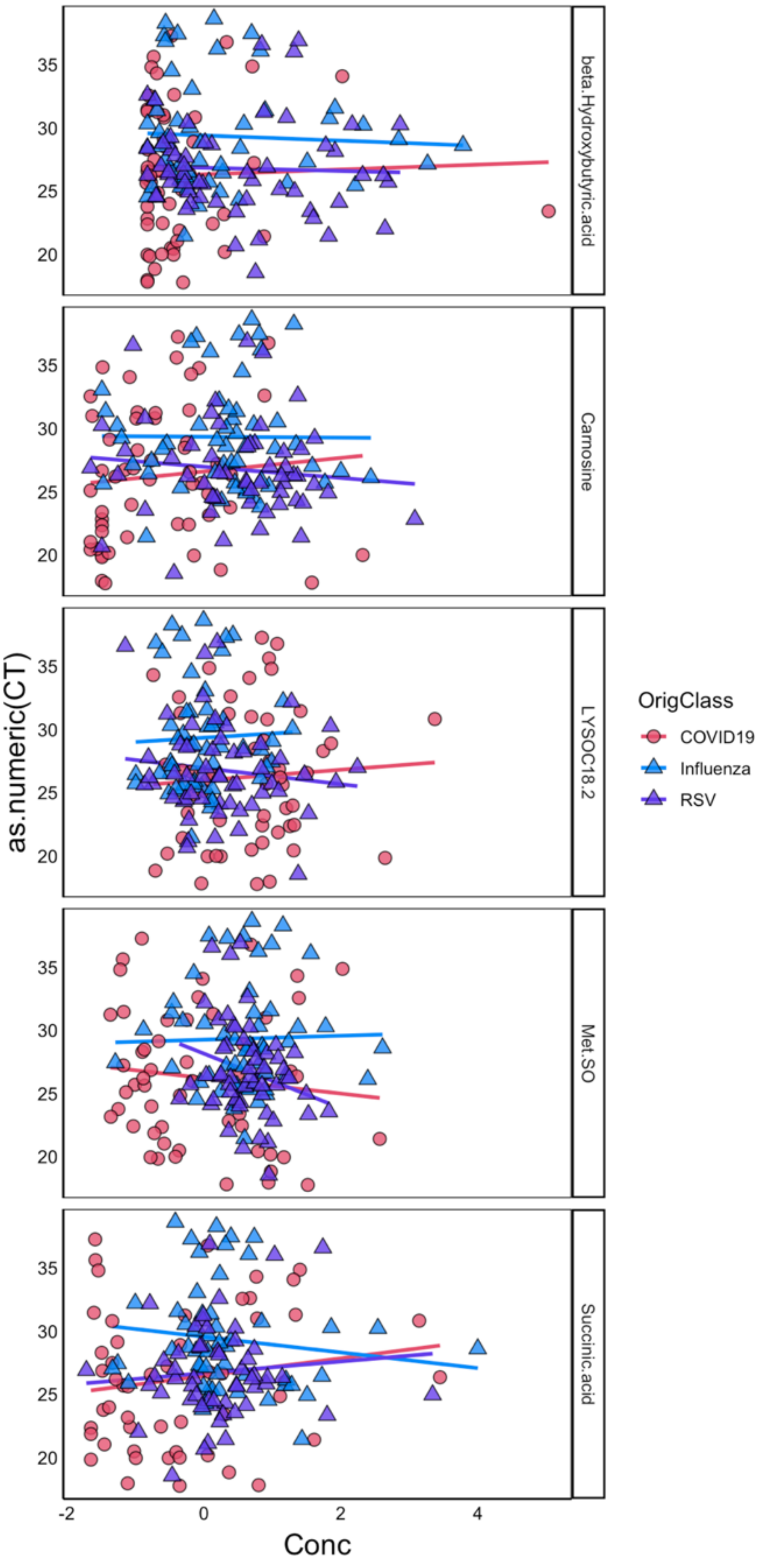

beta.Hydroxybutyric.acid
Carnosine
LYSOC18.2
Met.SO
Succinic.acid
as.numeric(CT)
Conc
OrigClass
COVID19
Influenza
RSV

Statistical tests

```
for(Met in unique(CTdat$Metab)){
  for(Cls in unique(CTdat$OrigClass)){
    StatDat<-CTdat[CTdat$Metab == Met &
                     CTdat$OrigClass == Cls,]
    print(anova(lm(Conc~as.numeric(CT), data=StatDat)))
    StatDat<-NA
  }
}
```

```
## Analysis of Variance Table
##
## Response: Conc
##                Df Sum Sq Mean Sq F value Pr(>F)
## as.numeric(CT)  1  1.857   1.857  1.2914  0.261
## Residuals      52 74.775   1.438
## Analysis of Variance Table
##
## Response: Conc
##                Df Sum Sq Mean Sq F value Pr(>F)
## as.numeric(CT)  1  0.748 0.74751  0.8974  0.348
## Residuals      50 41.649 0.83298
## Analysis of Variance Table
##
## Response: Conc
##                Df  Sum Sq Mean Sq F value Pr(>F)
## as.numeric(CT)  1  0.2763 0.27626  0.4752 0.4936
## Residuals      53 30.8101 0.58132
## Analysis of Variance Table
##
## Response: Conc
##                Df Sum Sq Mean Sq F value Pr(>F)
## as.numeric(CT)  1  0.325 0.32495  0.4108 0.5244
## Residuals      52 41.133 0.79101
```

```
## Analysis of Variance Table
##
## Response: Conc
##                 Df Sum Sq Mean Sq F value Pr(>F)
## as.numeric(CT)  1  0.002 0.00228  0.0029 0.9572
## Residuals      50 39.210 0.78421
## Analysis of Variance Table
##
## Response: Conc
##                 Df Sum Sq Mean Sq F value Pr(>F)
## as.numeric(CT)  1  0.612 0.61189  0.6768 0.4144
## Residuals      53 47.918 0.90411
## Analysis of Variance Table
##
## Response: Conc
##                 Df Sum Sq Mean Sq F value Pr(>F)
## as.numeric(CT)  1  0.648 0.64801  0.6642 0.4188
## Residuals      52 50.733 0.97564
## Analysis of Variance Table
##
## Response: Conc
##                 Df  Sum Sq Mean Sq F value Pr(>F)
## as.numeric(CT)  1  0.0166 0.01662  0.0333 0.8559
## Residuals      50 24.9443 0.49889
## Analysis of Variance Table
##
## Response: Conc
##                 Df Sum Sq Mean Sq F value  Pr(>F)
## as.numeric(CT)  1 0.6493 0.64928  3.5978 0.06331 .
## Residuals      53 9.5646 0.18046
## ---
## Signif. codes:  0 '***' 0.001 '**' 0.01 '*' 0.05 '.' 0.1 ' ' 1
## Analysis of Variance Table
##
```

```
## Response: Conc
##                  Df Sum Sq Mean Sq F value Pr(>F)
## as.numeric(CT)  1  0.137 0.13702  0.2065 0.6515
## Residuals      52 34.512 0.66368
## Analysis of Variance Table
##
## Response: Conc
##                  Df  Sum Sq  Mean Sq F value Pr(>F)
## as.numeric(CT)  1  0.0228 0.022824  0.0829 0.7746
## Residuals      50 13.7639 0.275278
## Analysis of Variance Table
##
## Response: Conc
##                  Df  Sum Sq Mean Sq F value Pr(>F)
## as.numeric(CT)  1  0.4731 0.47306  0.8661 0.3562
## Residuals      53 28.9470 0.54617
## Analysis of Variance Table
##
## Response: Conc
##                  Df Sum Sq Mean Sq F value Pr(>F)
## as.numeric(CT)  1  0.049 0.04939  0.0569 0.8124
## Residuals      52 45.145 0.86817
## Analysis of Variance Table
##
## Response: Conc
##                  Df Sum Sq Mean Sq F value Pr(>F)
## as.numeric(CT)  1  0.173  0.1728  0.1397 0.7102
## Residuals      50 61.866  1.2373
## Analysis of Variance Table
##
## Response: Conc
##                  Df Sum Sq Mean Sq F value Pr(>F)
## as.numeric(CT)  1  0.086 0.08638  0.0729 0.7883
## Residuals      53 62.828 1.18544
```

Stats summary: Influenze has significantly higher CT count overall, but no effect of

What about COVID1 axis from OPLS-DA model – does it predict CT values?

Setup:

```
COVIDmet<-c("Carnosine","Met.SO","beta.Hydroxybutyric.acid",
            "LYSOC18.2","Succinic.acid")
CTcomp<-COVIDdat[,COVIDmet]

CTcomp$estCOVID1<-rowSums(t(OPLSMod2@loadingMN[,1]*t(CTcomp)))

CTcomp$Sample<-rownames(CTcomp)
CTcomp$CT<-COVIDdat$CT
CTcomp$OrigClass<-COVIDdat$OrigClass
pDat2$Sample<-rownames(pDat2)

if(flipCOVID==T){
  pDat2$COVID1<-pDat2$COVID1*-1
}

pDat3<-full_join(pDat2[,c("Sample","COVID1")],
                        CTcomp[,c("Sample","estCOVID1","OrigClass","CT")],by="
Sample")
```

Double-check proper calculation of COVID1 in full dataset

```
qplot(x=estCOVID1,y=COVID1,data=pDat3)
```

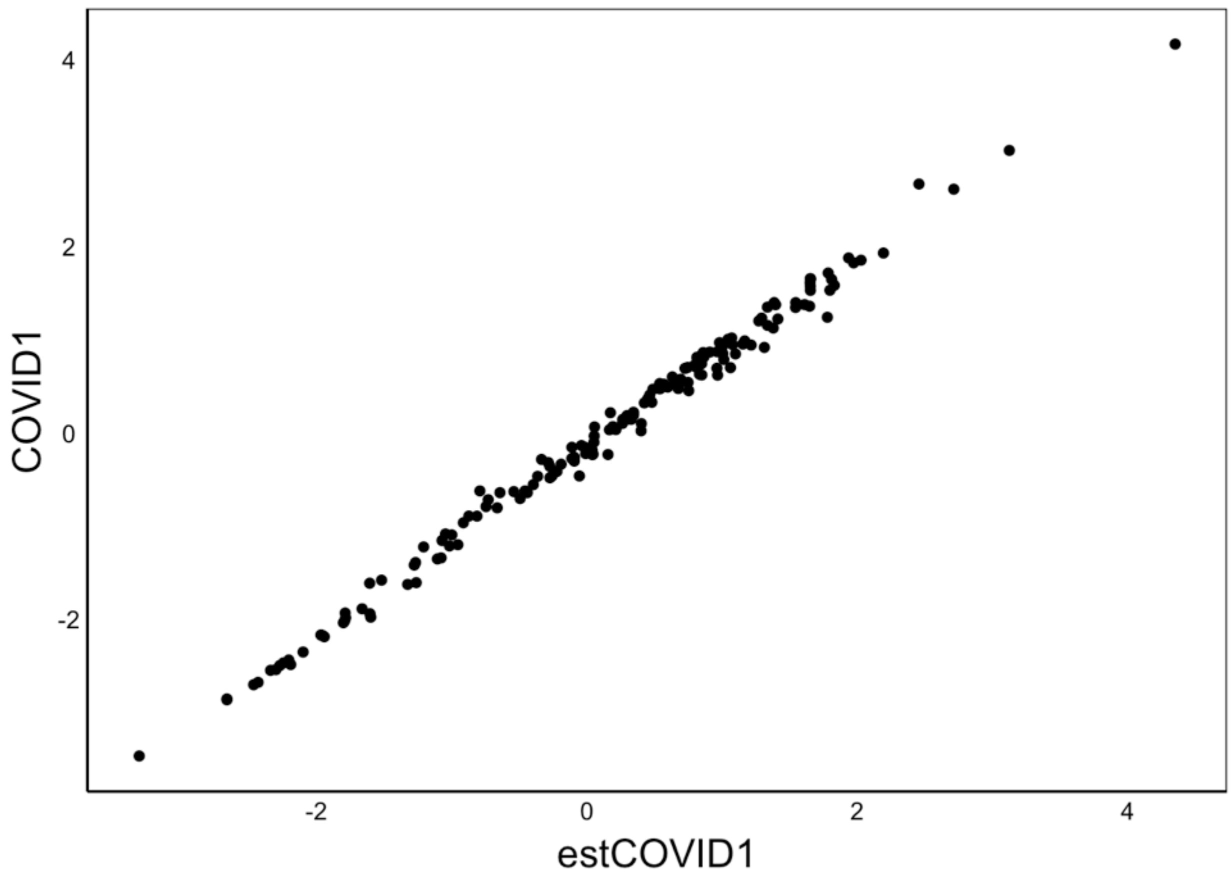

Test for CT correlation

```
ggplot(aes(x=estCOVID1,y=as.numeric(CT)),data=pDat3) +
  geom_smooth(aes(group=OrigClass,colour=OrigClass),se=F,method="lm") +
  geom_point(aes(group=OrigClass,fill=OrigClass,shape=OrigClass),
             size=3,alpha=0.8) +
  scale_fill_manual(values=c("#E54F6D","#008BF8","#623CEA")) +
  scale_colour_manual(values=c("#E54F6D","#008BF8","#623CEA")) +
  scale_shape_manual(values=c(21,24,24))
## `geom_smooth()` using formula 'y ~ x'
## Warning: Removed 5 rows containing non-finite values (stat_smooth).
## Warning: Removed 5 rows containing missing values (geom_point).
```

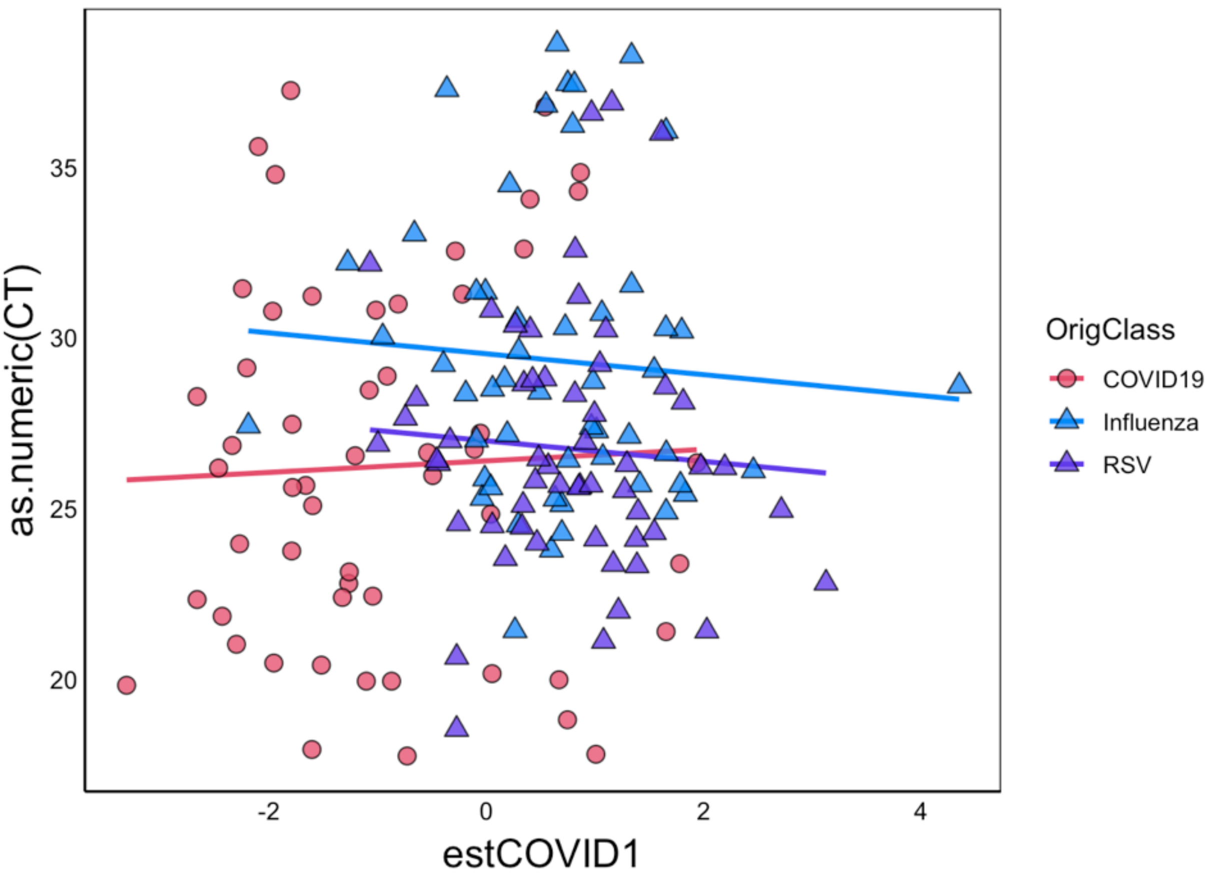
as.numeric(CT)
35
30
25
20
-2
0
2
4
estCOVID1
OrigClass
COVID19
Influenza
RSV
